# Dynamic eco-techno-economic analysis of low-carbon hydrogen production from methane

**DOI:** 10.1039/d5ya00346f

**Published:** 2026-02-02

**Authors:** Giulio Martinoli, Emanuele Moioli

**Affiliations:** a Dipartimento di Chimica, Materiali e Ingegneria Chimica ‘Giulio Natta’, Politecnico di Milano Piazza Leonardo da Vinci 32 20133 Milano Italy emanuele.moioli@polimi.it; b Center for Energy and Environmental Science, Paul Scherrer Institut Forschungsstrasse 111 5232 Villigen Switzerland

## Abstract

Hydrogen is currently produced predominantly through fossil fuel reforming, which accounts for approximately 3% of annual global CO_2_ emissions. To reduce the carbon intensity of hydrogen production, several low-carbon alternatives have been proposed, including biogas reforming and electrified steam methane reforming (e-SMR). Biogas benefits from its biogenic origin, leading to near net-zero carbon emissions, while e-SMR replaces the natural gas combustion used for reactor heating in conventional SMR with electrical heating. This modeling study performs a dynamic techno-economic assessment of these processes in comparison with state-of-the-art steam methane reforming (SMR) and auto-thermal reforming (ATR), evaluating the impact of implementing carbon capture and permanent storage (CCS). The analysis incorporates time-resolved and seasonal variations of real electricity prices in French, Swiss and German scenarios, employed as reference cases for low and high electricity grid footprints. Large-scale SMR and ATR plants exhibit the highest process efficiency (79–81%), which remains stable when CCS is implemented (77–81%). Lower efficiencies are observed for biogas reforming (56–67% with base case and 65–69% with CCS) and e-SMR (59% with base case and 71% with CCS) due to their smaller scale and the presence of CO_2_ in the feed. CCS significantly reduces carbon footprints: from 8.6–8.7 to 1.2–3.4 kg_CO_2__ kg_H_2__^−1^ for SMR and ATR and from 0.2–1.0 to −10 to −4 kg_CO_2__ kg_H_2__^−1^ for biogas reforming. e-SMR emissions (from 6–18 to 0.3–10 kg_CO_2__ kg_H_2__^−1^ with CCS) depend strongly on the electricity mix. The possible presence of carbon credits makes the application of CCS economically beneficial for SMR and ATR (H_2_ cost ranging from 1.6 to 1.3 € per kg_H_2__) and for biogas reforming (from 3.7 to 3.5 € per kg_H_2__). e-SMR competitiveness is highly electricity-price-dependent and benefits from CCS regardless of carbon credits, performing best in France (3.7 to 2.6 € per kg_H_2__ with CCS) and worst in Switzerland (4.2 to 3.1 € per kg_H_2__ with CCS). Intermittent operation to exploit low-cost electricity may further reduce e-SMR costs by 0.1–0.4 € per kg_H_2__.

## Abstract 2

This study performs an eco-techno-economic analysis of hydrogen production from steam reforming, biogas reforming and electrified reforming with and without CO_2_ capture, including the dynamic effects of green electricity availability and cost.

## Graphical abstract

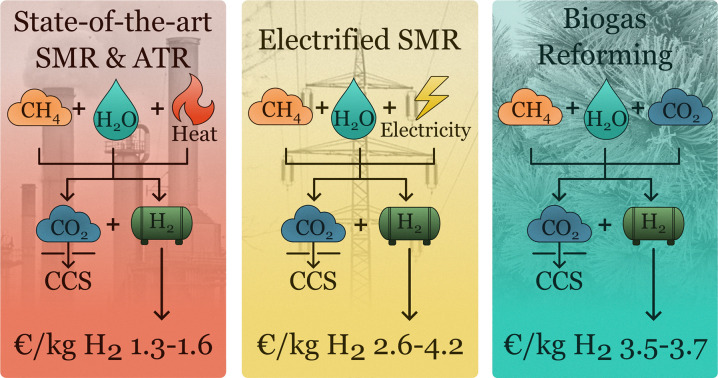

## 1 Introduction

Hydrogen is expected to play a key role in the near future as an energy carrier in all hard-to-abate industrial sectors, as the targets of the Paris Agreement aim to limit global warming to 1.5 °C or 2 °C and not all energetic applications can be directly electrified.^1,2^ Although water electrolysis has received great attention in the scientific literature for hydrogen production, reforming fossil fuels remains the dominant industrial pathway. Natural gas accounts for 48% of the overall H_2_ demand, oil for 30% and coal for 18%, while water electrolysis covers only a scarce 4% of the global H_2_ supply.^3–5^ H_2_ production is responsible for approximately 3% of global CO_2_ emissions, due to fuel combustion required by conventional fossil-based processes.^6^ The standard natural gas reforming process is strongly endothermic and proceeds through the reactions of steam methane reforming and water gas shift:1
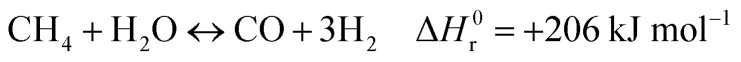
2
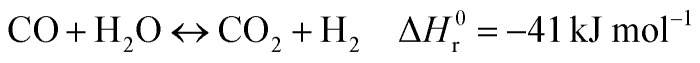
where Δ*H*^0^_r_ is the standard reaction enthalpy. In this conventional process, heat supply is usually provided by combusting a mixture of natural gas (which acts as both a feed and a fuel) and off-gas from the product stream, contributing to 17–41% of the 6.6–9.3 metric tons of CO_2_ emitted per metric ton of H_2_.^6,7^ The high carbon footprint of the state-of-the-art gray hydrogen, along with the urgent need to establish hydrogen as a key energy vector, highlights the importance of implementing new low-carbon production pathways for H_2_ in the near future.

### 1.1 Existing technologies for low-carbon hydrogen: literature review

Water electrolysis is one of the most encouraging and widely studied ways for the production of green H_2_, especially when excess renewable electricity is exploited.^8^ However, the carbon footprint of electrolysis is strongly dependent on the energy source adopted by the process, which determines the indirect carbon emissions of the product.^3,9–12^ Moreover, the cost of hydrogen from electrolysis is bound to the price of electricity and, in most cases, is higher than the benchmark.^13^ In addition to water electrolysis, a low-carbon process for hydrogen production is methane pyrolysis, which converts methane to H_2_ and solid carbon. Although promising for future applications, this pathway is yet to have a satisfactory technology readiness level (TRL), due to the complexity in scale-up.^14^ The use of biogas instead of natural gas as feedstock for the reforming process is an interesting alternative for the geographically distributed production of H_2_ or syngas, as assessed in various works.^15,16^ However, the limited size of biogas plants affects the overall maximum achievable production efficiency. Biomass is commonly regarded as a carbon-neutral energy resource, as CO_2_ emissions released during its processing and combustion are generally considered to be offset by CO_2_ uptake through photosynthesis during plant growth.^17^ Consequently, in other works, biogenic CO_2_ emissions are often assigned a global warming potential (GWP) of zero.^18–20^ This makes biogas a viable candidate for low-carbon reforming. Another feasible route for low-carbon hydrogen production consists of combining either natural gas or biogas reforming with carbon capture and storage (CCS), referred to as blue hydrogen. Several technologies enable CO_2_ removal from the produced syngas, such as chemical absorption, chemical adsorption, membranes and cryogenic processes; various works assessed the techno-economics of blue hydrogen, highlighting an increase of roughly 20–60% in the cost of hydrogen compared to the benchmark, accompanied by the capture of around 55–90% of the produced CO_2_.^15,21–24^ Moreover, in the case where biogenic emissions as those of biogas are permanently stored, this contribution is accounted for as a negative emission pathway.^25^ The use of an electrified reformer, consisting of electric resistances or induction-based devices for the heat supply to the reactor, was investigated in several works. These consider adopting methane or biogas as feedstock and demonstrated that the compactness of this layout can enhance the heat transfer efficiency between the heating medium and the catalyst core.^26–28^ Moreover, From *et al.*^27^ evidenced how the use of a scaled up process would even improve the performance of the conductive devices due to the reduction in the specific surface area, leading to lower heat losses. Replacement of natural gas combustion with electrical heating removes a large fraction of CO_2_ emissions in gray hydrogen production, but bounds the carbon footprint of the product to the exploited energy source, similar to the case of water electrolysis.

### 1.2 Scope of this work

This work consists of a detailed techno-economical and environmental comparison of natural gas reforming, biogas reforming, and electrified methane reforming. Each process is modeled with and without the implementation of carbon capture and storage, to underline the benefits and drawbacks of existing benchmark technologies for CO_2_ removal in industrial processes, in terms of plant performance, carbon emissions and economic metrics. Several exhaustive techno-economical studies on hydrogen production are present in the literature,^15,29–32^ but the dynamic nature and regional variation of electricity cost are usually not taken into account in the levelized cost of hydrogen (LCOH) assessments, which typically rely on fixed electricity prices derived from recent national observations (*e.g.* yearly averaged values) resulting in a static evaluation. The need for a dynamic analysis arises from the volatility of the electricity cost in many competitive markets around the world, due to the demand for electricity, the availability of generation sources, the fluctuation in fuel costs, and the availability of power production plants.^33^ This element is of particular importance for electrified processes, where the cost of electricity plays an important role in determining the economic performance of the process. This work includes the evaluation of the influence of geographical and temporal variability of the electricity cost and footprint on the final cost of hydrogen in real case scenarios. This work addresses the lack of a region-specific, dynamic LCOH evaluation by performing it alongside a steady-state LCOH assessment based on time-averaged electricity prices. Both analyses use day-ahead electricity price time series from January 2023 to October 2025 for France, Germany, and Switzerland, obtained from the EPEX SPOT database.^34^ The dynamic study is particularly crucial for electrified steam methane reforming, which is the main technology investigated in this work, whose start-up time is significantly shorter than that of large-scale, fossil-based reforming. This property of the system allows the plant to operate when the most favorable electricity prices occur and to shut down it under unprofitable conditions. In this work, a specific time-dependent scenario is developed for electrified steam methane reforming, according to what was discussed above. The final scope of the whole investigation consists, for each technology, in assessing the plant efficiency (*η*), the process-related carbon footprint (CFP) and the levelized cost of hydrogen (LCOH).

## 2 Methods

**Fig. 1:**
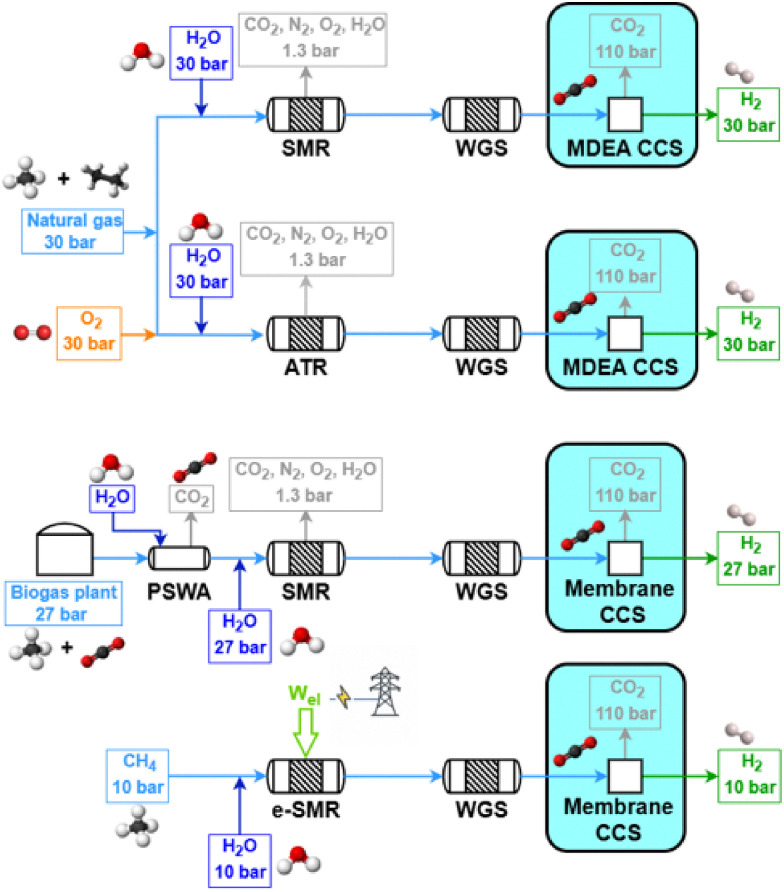
Visualization of the plant battery limits. The CCS unit is highlighted, as it is optional in the plant layout.

The technical analysis presented in this work consists of simulating all processes in MATLAB R2025b through the resolution of mass and energy balances at the plant scale with the fsolve solver function, aiming at the assessment of specific technical, economic, and environmental metrics. The following assumptions and system boundaries are established for the design of the different processes:

(1) Gaseous streams are modeled as ideal gas and ideal mixtures;

(2) enthalpy is modeled consistent with ref. 35 using temperature-dependent polynomial correlations (third-order for liquid phase enthalpy and fourth-order for gas phase enthalpy);

(3) heat losses are assumed to be negligible for reactors (except for electrified reforming) and for heat recovery sections, and chemical equilibrium is assumed to be reached at the reactor outlet;

(4) pressure drops are considered negligible;

(5) the system boundaries are defined as reported in Fig. 1;

(6) in electrified steam methane reforming and biogas reforming, it is assumed that the feedstock is already desulfurized;

(7) compression duties, when required, are estimated using Aspen HYSYS v7.3, except for SMR and ATR schemes, whose CO_2_ compression is based on the literature;^36^

(8) pumping duties are neglected, whereas vacuum pump duties are estimated based on literature data;^15^

(9) water condensation is simulated as producing pure water with a 100% recovery efficiency;

(10) hydrogen separation through VPSA/PSA is simulated as producing 100% pure hydrogen, consistent with 99.99% purities observed in other works;^29,36^

(11) carbon removal is performed prior to the PSA/VPSA unit for hydrogen purification, the so-called “pre-combustion” CO_2_ capture. Previous works pointed to this choice as better from both an energetical and an economical point of view, compared to post-combustion CO_2_ capture;^21,29,37^

(12) the implementation of a carbon capture unit leads to the production of 100% pure CO_2_, which is subsequently compressed to 110 bar and stored in pressurized storage vessels;

(13) the use of captured CO_2_ after compression is beyond the scope of this work; utilization in downstream processes (*e.g.* urea or methanol production) or permanent geological storage is assumed to be handled by third parties.

Furthermore, the selected process variables for the technologies of this study, whose choice is discussed in the following sections, are summarized in Table A.7. Economic analysis includes both investment and operating costs. The evaluation is based on the literature^38–40^ and is carried out through scaling factors. The dynamic analysis of the electricity price is based on the EPEXSPOT database.^34^

### 2.1 Summary of the technologies investigated

A summary of the technologies investigated is reported in Table 1. Steam methane reforming (SMR) and auto-thermal reforming (ATR) are selected as benchmark technologies for large scale fossil-hydrogen production, collectively accounting for approximately 48% of the global H_2_ demand. In these processes, heat supply for the methane reforming reaction relies on fuel combustion, which is performed, respectively, through external firing of the tubular reactor for SMR, and directly in the vessel-sized reactor for ATR. A similar approach to the SMR heating system is used for biogas steam reforming (BSR), whose main difference, aside from the feedstock, consists of the smaller scale of the process, which is due to the reduced and delocalized availability of biogas compared to natural gas.^15^ Fuel combustion is replaced by the use of electrical heating through the Joule effect in electrified steam methane reforming (e-SMR) technology, which is implemented on a closer scale to BSR rather than to SMR. Besides state-of-the-art technologies for fossil hydrogen production, benchmark routes for low-carbon hydrogen are aimed at a comparison with the investigated BSR and e-SMR. These are represented by alkaline water electrolysis (AWE), aimed at splitting water into hydrogen and oxygen through electricity, and methane pyrolysis (MP), involving the decomposition of methane into hydrogen and atomic carbon with the use of a direct electric heating. Different from reforming-based processes, AWE and MP are not directly modeled and simulated within this work, rather reference values from previous works are used for the comparison.

**Table 1:** Summary of the technologies investigated

Acronym	Full name	Heat source	Feedstock
SMR	Steam methane reforming	External firing from fuel combustion	Natural gas
ATR	Auto-thermal reforming	Internal fuel combustion	Natural gas
BSR	Biogas steam reforming	External firing from fuel combustion	Biogas
e-SMR	Electrified steam methane reforming	Direct electric heating *via* the Joule effect	Methane
AWE	Alkaline water electrolysis	—	Water and electricity
MP	Methane pyrolysis	Direct electric heating *via* the Joule effect	Methane

### 2.2 Natural gas reforming

The performance evaluation of natural gas reforming is essential in this work, as it defines a benchmark for comparing novel technologies. The feed, natural gas, is assumed to have a composition similar to that in the study by Antonini *et al.*,^36^ except for the molar fraction of *n*C_4_H_10_, which is removed for simplicity due to the negligible impact on simulations. In addition to that, carbonyl sulfide is added to the composition based on ref. 41, for a better description of the impurities in the feed.

Two different types of reforming technologies are investigated. The classification is based on the type of reactor: fired tubular steam methane reforming (SMR) and auto-thermal reforming (ATR).

#### 2.2.1 Fired tubular natural gas reforming

**Fig. 2:**
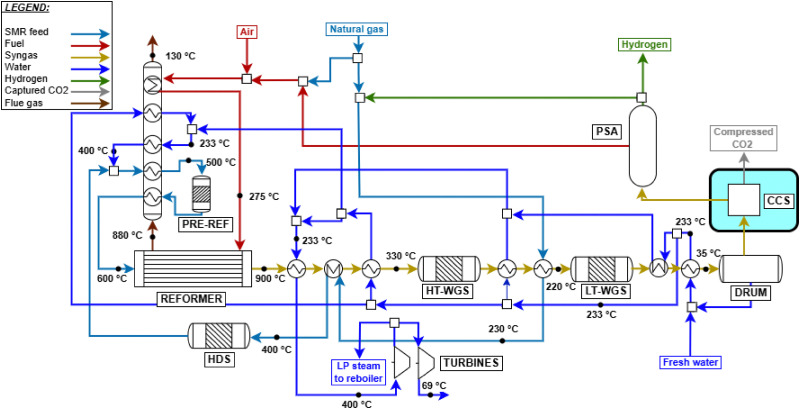
Natural gas steam reforming plant scheme with HT + LT-WGS and CCS units, whose addition is highlighted.

The fired tubular natural gas reformer represents the state-of-the-art technology for hydrogen production and serves as the upstream process for large-scale commodities such as ammonia and methanol. The process configuration adopted in this study is based on previous works^29,36^ and is optimized to identify the optimal working conditions. The process scheme is shown in Fig. 2. Natural gas at 30 bar (operating pressure of the plant, *P*) is divided into two streams: a feed stream to the reformer tubes (*F*_NG,ref_, molar flowrate) and a fuel stream for the furnace (*F*_NG,fuel_). This defines the splitting factor, *α*, as3
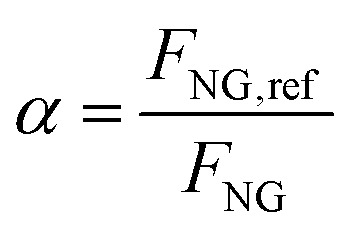
where *F*_NG_ is the total natural gas feed to the plant battery limits, set to 1700 kmol h^−1^ according to the literature.^29,36^ The stream *F*_NG,ref_ is preheated to 400 °C using process-to-process heat exchangers and subsequently fed to the hydrodesulphurization (HDS) unit. The HDS unit usually consists of two catalytic beds operating at 350–400 °C:^41^ the first ensures complete hydrogenation of sulfur compounds to H_2_S, while the second adsorbs the produced H_2_S to prevent poisoning of the steam reforming catalyst. A small fraction of the produced hydrogen is recycled to the HDS unit to promote sulfur conversion:

4 COS + H_2_ → CO + H_2_S

5 ZnO + H_2_S → ZnS + H_2_O

The desulfurized natural gas is then mixed with steam. Steam generation relies on process-to-process heat recovery, defined through a pinch analysis. The steam flowrate *F*_w_ is characterized by the steam-to-carbon ratio (S/C):6
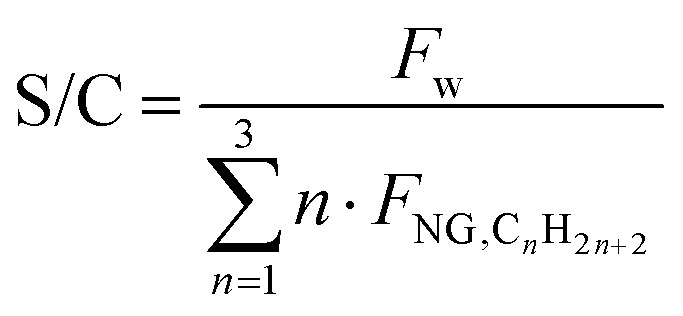
where *F*_NG,C_*n*_H_2*n*+2__ represents the molar flowrate of each hydrocarbon in the natural gas feed. As in the literature, S/C is defined as the ratio of steam moles to carbon moles excluding CO_2_^42^ and is set to 2.7 (optimum between energy requirements and the catalyst lifetime). The mixture is then overheated to 500 °C and fed to the pre-reformer, designed to convert C_2+_ hydrocarbons (in this case, ethane and propane) by catalytic reforming:7
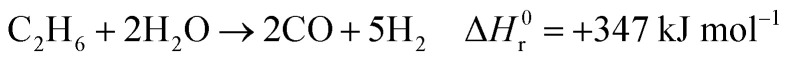
8
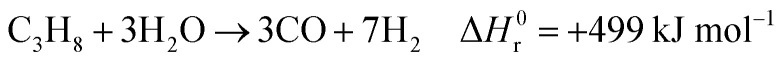


As the pre-reformer operates adiabatically, these endothermic reactions decrease the temperature of the flow, requiring subsequent reheating to 600 °C before entering the main reformer. This inlet temperature minimizes the thermal duty at the reactor by maximizing process heat recovery and is consistent with values commonly reported in the literature.^29,36,43,44^ The externally fired tubular reformer constitutes the core of the process, and its structure resembles a heat exchanger with catalyst-filled tubes.^41^ The reacting gas flows inside the tubes, while the furnace provides heat by both radiation from the combustion flames and convection from the flue gas. Combustion is complete, using preheated natural gas, tail gas, and air at 275 °C, with 7% excess air.^45^ The flue gas leaves the reformer at 880 °C, consistent with industrial ranges of 800–900 °C^29^ and with values adopted in previous works.^45,46^ The reformer outlet temperature (*T*_out_) is set to 900 °C, and the reformer effluent (syngas) consists mainly of H_2_, CO, CO_2_, and unconverted CH_4_. The stream is cooled to 310 °C *via* successive heat exchangers and sent to one or two adiabatic water–gas shift (WGS) reactors, depending on the required CO conversion. The first reactor, or high-temperature WGS (HT-WGS), operates adiabatically at 310 °C (no steam addition is foreseen), within the 310–450 °C range suitable for the catalyst operation^47^ and achieves 65–70% CO conversion. Inlet temperature is decreased as low as allowed by reaction kinetics to enhance the equilibrium conversion (see Fig. A.3). The temperature increases along the reactor due to the heat production caused by the reaction. For high-purity hydrogen production (*e.g.*, for downstream ammonia synthesis), a second reactor, the low-temperature WGS (LT-WGS), is added. Here, the inlet temperature is set to 210 °C, within the optimal 210–240 °C range for catalyst performance,^47^ aimed at the achievement of around 90% CO conversion at equilibrium. The use of both HT and HT + LT water–gas shift reactors is analyzed in two distinct configurations for the SMR process. After the WGS section, the gas is cooled to 35 °C and sent to a condensation drum to remove condensed water. The resulting dry gas is then directed either to a pressure swing adsorption (PSA) unit for hydrogen purification or to a carbon capture unit, depending on the process configuration. This translates in two distinct plant layouts, with and without the use of CCS, for each of the two designed SMR configurations (HT-WGS and HT + LT-WGS). When carbon capture is included, CO_2_ absorption using MDEA is adopted as the benchmark technology. The model of the unit is based on the work of Antonini *et al.*, 2021,^48^ consisting in the use of two sections for, respectively, carbon dioxide absorption (*P* = 30 bar and *T* = 35 °C) and desorption (*P* = 1.15 bar). MDEA solution is assumed to have c/m (CO_2_ to MDEA molar ratio) = 0.3 and *w*_M_ (MDEA weight fraction in CO_2_-free solution) = 0.48. The capture efficiency is set to 90%, and the electric consumption for CO_2_ compression (from desorption pressure to 110 bar) and utilities is assumed to be 0.42 MJ t_CO_2__^−1^, while the reboiler duty is 0.78 MJ_th_ t_CO_2__^−1^. The heat duty to the reboiler is required for the regeneration of the amine solution through desorption. To this purpose, no external heat sources, for boiling feed water (BFW), are employed; instead, heat is supplied by the condensation of low-pressure saturated steam (133 °C, 3 bar) extracted from the first turbine stage,^32^ whose definition is reported below. The compression work calculated aligns with the values reported by Cormos^37^ for pre-combustion CCS. The PSA unit comprises a series of adsorption beds, operated under pseudo-steady-state conditions, where impurities (CO, CO_2_, and CH_4_) are adsorbed, while the majority of hydrogen is purified and recovered in the passing gas stream. The hydrogen recovery efficiency is set to 90%, which is an industrially feasible target according to the study by Luberti *et al.*^49^ This value of efficiency agrees with other sources.^29,36,38^ Finally, the purified hydrogen at 30 bar is partly recycled (0.5%) to the HDS unit as aforementioned, while the PSA tail gas at 1.3 bar (PSA desorption pressure based on ref. 29) is blended with fuel and air and combusted in the reformer furnace. Flue gas derived from the combustion leaves the reformer at 880 °C and passes through a heat recovery section to be cooled until 130 °C through process-to-process heat exchanges, which are properly designed as discussed for the syngas cooling section. This outlet value is higher than the mixture dew temperature at 1 atm, which is assessed within 50–55 °C depending on the case, avoiding water condensation in the heat recovery section. Syngas and flue gas cooling imply the release of a larger amount of heat required by the process. Hence, excess steam at 30 bar is produced and exploited for the generation of electricity using a two-staged steam turbine. The latter consists of two subsequent gas expansions, from 30 bar, 400 °C overheated steam to 3 bar, 133 °C saturated steam, to finally 0.3 bar and 69 °C saturated steam, by assuming isentropic and electrical efficiency as equal to, respectively, 94% and 95%, coherently with the study by Antonini *et al.*, 2020.^36^ As mentioned before, some of the adopted parameters (S/C, *T*_out_, *P*, and *T*_fuel_) are chosen through a sensitivity analysis to highlight their influence on *α* and on the LHV-based plant efficiency *η*_LHV_, whose definition is reported in Section 2.6.1. The analysis is based on the simulation of the HT-SMR plant under the initial hypothesis of *T*_fuel_ = 250 °C. Fig. A.4 and A.5 show *η*_LHV_ and *α* trends at different S/C, *T*_out_, and *P* values. Steam addition is proven to be detrimental to the process efficiency, due to the steep increase of the heat duty required for its overheating to the equilibrium temperature, which implies lower splitting factors. For this reason, S/C is set to 2.7, which is the lower bound of the range proposed by Antzara *et al.*^50^ Avoiding S/C ratios below the lower bound is key to prevent severe carbon formation on the catalytic surface.^41^ Although pressure negatively impacts the efficiency, a suitably high pressure is a beneficial to the reduction of the operating volumes, according to the ideal gas law. Additionally, although the use of hydrogen is not directly specified by this work, higher pressure in the plant helps reducing the compression costs for downstream hydrogen uses, as indicated by Heidlage *et al.*, 2017.^51^ The same applies in case a high pressure hydrogen storage is present downstream. A value of 30 bar is selected as a compromise between operating volumes and process efficiency, which is also adopted by other works.^44,52,53^ A detailed analysis of the results at the selected pressure and S/C reveals *α* values above 100% for a given equilibrium temperature range (below 850 °C), as shown in Fig. A.5. This is due to the excess amount of available energy for the plant configuration under study, which leads the temperature choice to 900 °C, a value that allows reasonable efficiency and split factor. Similarly, a *T*_fuel_ value equal to 275 °C is chosen, enabling both a reasonable plant efficiency (which grows proportionally to this variable) and an *α* ratio of about 90%, through which a suitable heat control of the reformer is attained as reported in Fig. A.6.

#### 2.2.2 Auto-thermal natural gas reforming

An auto-thermal reformer (ATR) consists of a burner, a combustion chamber, and a catalyst bed, all of which are contained in a singe unit.^41^ Unlike the previously discussed steam reformer, external heating sources are not foreseen by this configuration, as heat is directly provided by the combustion of natural gas with either oxygen or pure air,9
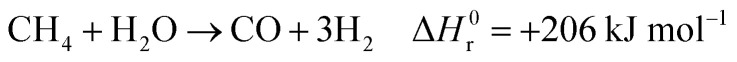
10
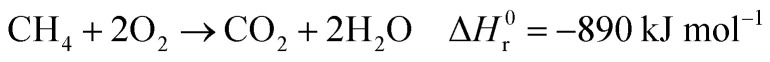


The quantity of O_2_ is defined as in eqn (6):11
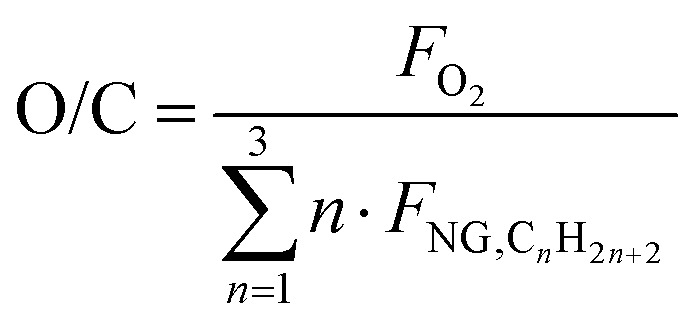
where *F*_O_2__ is the oxygen flowrate, and the remaining streams are defined as for the previous steam reforming plant. In this simulation, pure oxygen delivered from the air separation unit (ASU) is assumed as feed, together with natural gas and steam, consistent with the study by Antonini *et al.*, 2020.^36^ Air is usually fed in ammonia synthesis plants, as nitrogen acts directly as a reactant for the latter. However, pure O_2_ feed is considered here to obtain results that can be applied to any possible case. The plant scheme and assumptions are basically the same as those for the previous case; only a few adjustments are implemented:

(1) tail gas is sent to a burner, where heat is exploited both for reactant preheating and for the overheating of steam, which is used to attain power generation through steam turbines;

(2) the ATR inlet temperature is set to 700 °C based on the literature;^32^

(3) the ATR outlet temperature is determined on the basis of the quantity of oxygen added. This is evaluated as the equilibrium temperature of the steam methane reforming reaction, based on an energy balance assuming full oxygen conversion *via* methane combustion;

(4) the electricity duty in the ASU unit is assumed to be equal to 265 kWh t_O_2__^−1^ (based on the literature^36,54,55^).

**Fig. 3:**
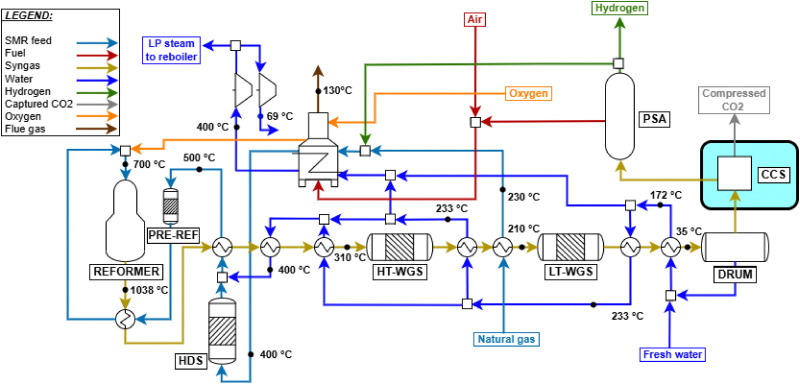
ATR plant process scheme with CCS, whose addition is highlighted.

Parametric analysis was performed to identify the optimal values for O/C, S/C and P, whose initial ranges are assumed within 0.4–0.6,^41^ 0.9–1.6^32^ and 25–40 bar, respectively. In addition to the previous metrics of *η*_LHV_ and *α*, the constraint of limiting the outlet temperature to 1050 °C is considered, as higher temperatures are not used in practice (due to limits on the stability of the catalyst and refractory lining^32,56^). As shown in Fig. A.7 both S/C and O/C have a beneficial impact on process efficiency, due to enhanced thermodynamic equilibrium conversion related to water addition and heat supply. Plant efficiency peaks around O/C = 0.56, and then the trend suddenly reverses, because a too large quantity of methane is combusted, limiting the amount of methane available for the steam reforming pathway. This assumption is demonstrated in Fig. A.8, as the reaction enthalpies of SMR (eqn (9)) and methane combustion (eqn (10)) are bounded by the stoichiometry of the two reactions, balancing at roughly O/C = 0.56 when *P* = 30 bar and *T*_out_ = 1050 °C. This temperature constraint leads to the choice of O/C = 0.56 and consequently S/C = 1.6. The pressure is set to 30 bar for the same reasons as for the previous SMR plant. When implemented, the CCS unit is positioned before tail gas combustion in the furnace, as in previous plant designs. Unlike in SMR plants, the specific layout of ATR also allows capturing CO_2_ originating from fuel combustion. The flow sheet of the designed plant is shown in Fig. 3.

### 2.3 Biogas reforming

Biogas is generated through anaerobic digestion of organic materials. Due to its biogenic source, it can be regarded as a circular raw material, helping to mitigate additional greenhouse gas emissions.^57^ The methane content in biogas depends on the nature of the feedstock (*e.g.*, agricultural biomass, municipal solid waste, or wastewater).^58^ Biogas usually consists of 55–58% methane and 37–38% carbon dioxide.^59^ The development of biogas-based chemical processes faces significant challenges, mainly because its production usually occurs in small-scale facilities that are often located in remote areas.^60^ As a result, biogas is most commonly utilized for electricity generation in combined heat and power (CHP) units. Nevertheless, the gradual reduction of incentives for electricity production and the difficulties associated with effective heat use have increased the interest in alternative biogas valorization routes.^61^ For these reasons, in this work, biogas from anaerobic digestion in isolated farms is considered as a possible feedstock for H_2_ production. The conversion process to hydrogen is similar to standard steam reforming. However, it must be implemented on a significantly smaller scale, as conventional biogas plants typically generate around 0.25 Nm^3^ s^−1^ of methane, whereas large-scale reforming facilities operate with flow rates of about 25–30 Nm^3^ s^−1^.^15^ Moreover, when CO_2_ is not removed from the feed to the reformer, the equilibrium conversion to H_2_ is less favorable than in previous configurations. Additionally, the presence of CO_2_ causes a less efficient hydrogen recovery, as the large amount of impurities in the system requires a different PSA/VPSA design compared to the one adopted for natural gas reforming.^15^ The smaller scale, combined with the larger presence of CO_2_ in the process, leads to some differences from previously defined plant configurations:

(1) pressures and outlet reactor temperatures are milder than those in previous processes;

(2) process-to-process heat exchangers are not foreseen as a result of the reduced heat duties;

(3) CCS is implemented through membranes, as the absence of available process heat burdens the operation of the MDEA process;^62^

(4) membrane technology is modeled consistent with the study by Lin *et al.*,^63^ achieving 90% CO_2_ recovery efficiency, and it is assumed that it does not require electrical work;

(5) the feedstock biogas (*F*_BG_) is assumed to have a molar composition of 55% CH_4_ and 45% CO_2_. This is the lower limit of the methane concentration,^59^ assuming that no other impurities than CO_2_ are present.

**Fig. 4:**
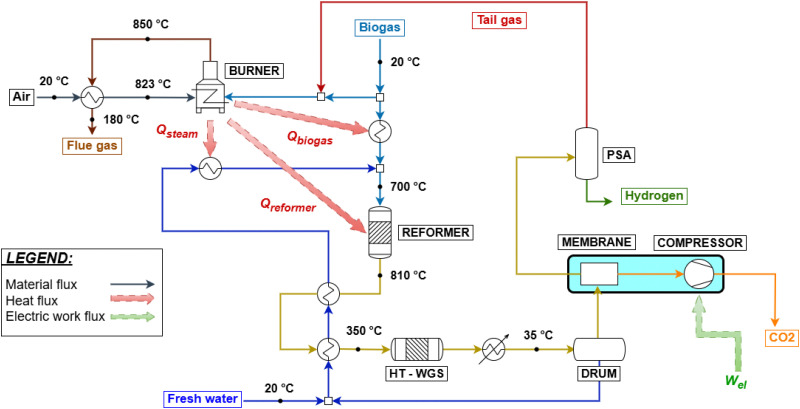
Biogas steam reforming through FLOX® with the CCS unit and PSA hydrogen separation.

**Fig. 5:**
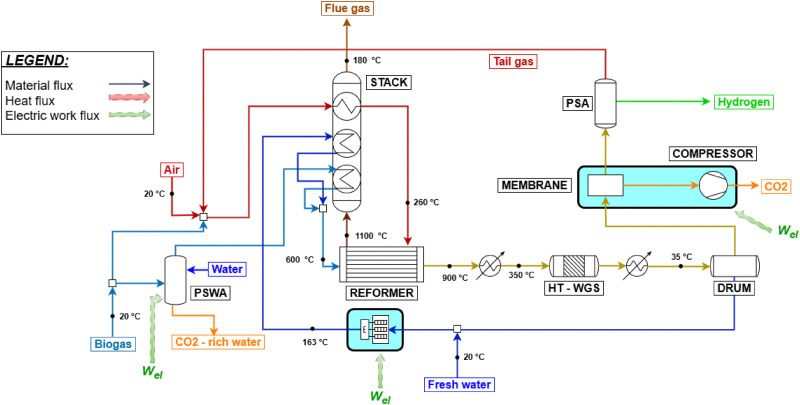
Biogas steam reforming with the PSWA biogas scrubbing and CCS unit.

Given these premises, two different configurations for hydrogen production from biogas are simulated: a compact steam reforming technology, FLOX®,^64^ and a small scale plant with the implementation of biogas scrubbing for the removal of CO_2_. FLOX® is a patented technology for small scale steam reforming, capable of overcoming the challenges related to the smaller process size, such as exhaust gas and reformate temperature losses, stable burner operation, easy electrical control and thermal coupling.^65^ The easier control is due to the adoption of flameless oxidation in the furnace.^66^ Due to the specific design, process-to-process heat couplings are achieved in accordance with the study by Schmid *et al.*,^64^ without the use of heat exchangers, which are possibly not implemented as discussed. The flow sheet of the process is reported in Fig. 4 and is similar to what was reported for natural gas reforming, except for the thermal recovery of hot streams. Biogas is divided into two streams feeding the burner and the reformer, respectively. The burner is coupled in such a way that biogas combustion with 5% excess air^67^ (which is preheated by flue gas cooling from 850 to 180 °C) supplies heat for the reforming reaction, biogas preheating to 700 °C and steam overheating to 700 °C, respectively. Syngas produced at 810 °C is cooled to 350 °C by vaporizing feed water and is used in a HT-WGS reactor. The shifted stream is condensed through a utility-based cooler, water is removed, and hydrogen is eventually purified through VPSA (vacuum pressure swing adsorption). The modeling of a VPSA instead of conventional PSA is based on the literature^15^ and is required due to the large presence of CO_2_ at the separator inlet (almost the 30% molar basis, against 17% of conventional SMR/ATR processes). This affects the recovery of hydrogen to a scarce 60%. As no specific use for tail gas is foreseen by technology owners,^64^ it is assumed to be re-compressed from VPSA desorption pressure (0.1 bar) to 1.5 bar and recycled to the burner. Finally, in case CCS is implemented, the PSA unit is used instead of VPSA, as CO_2_ content in the product stream is reduced in the membrane separation step, resulting in a composition similar to the SMR case. In fact, although they may be expected to be economically challenging, small-scale PSA units for hydrogen purification are available.^68^ As a result, the tail gas desorption pressure increases from 0.1 bar to 1.3 bar, eliminating the need for an additional compressor and simplifying the overall system design. The process variables are set based on the literature.^64,65^ The high S/C ratio results from the need to prevent coke formation for the given compact layout, as reported by Salano *et al.*, 2024 (ref. 65). As an alternative, the exploitation of a pressure swing water adsorption (PSWA) unit for CO_2_ removal from biogas is assessed for a biogas steam reforming plant. The process flow sheet is roughly the same as in SMR hydrogen production, except from the heat exchangers and the biogas cleaning unit. This is shown in Fig. 5. Additionally, in comparison with the FLOX® process, no process-to-process heat couplings are envisaged, except for heat recovery within the heat recovery section. This is feasible because no actual heat exchangers are employed for the latter, but rather simple tubes running through the heat recovery section. Salano *et al.*^67^ investigated this approach for methanol production from syngas, demonstrating that the most economically favorable option is to scrub only the biogas stream intended to feed the reformer, rather than treating the fraction designated for fuel use. This result is the basis for the defined process design. The consumption of the PSWA unit in terms of electricity and water is set in accordance with ref. 69, with the goal of producing an upgraded biogas containing 5 mol% CO_2_, which is identified as the optimal value to minimize both water consumption and CO_2_ content. Due to the biogenic origin of biogas, CO_2_ is vented after being separated from water. The reformer and the HT-WGS are modeled similar to the SMR process in terms of input and outlet temperatures. A LT-WGS unit is not included, due to the smaller scale of the plant. The PSA unit is modeled the same as for the previous schemes. This is made possible by the initial CO_2_ removal. Tail gas is fed as fuel to the reformer, while the flue gas heat recovery section is designed to cool the gas from 1100 °C to around 130 °C. The CCS unit is modeled as in the previous case. An electric heater is added to the water vaporization section, to have a flue gas outlet temperature equal to 180 °C as assessed by pinch analysis. The biogas feed rate is 500 Nm^3^ h^−1^, chosen to approximate the average Italian plant size of 1 MW_el_ equivalent, as reported in ref. 70.

### 2.4 Electrified methane reforming

**Fig. 6:**
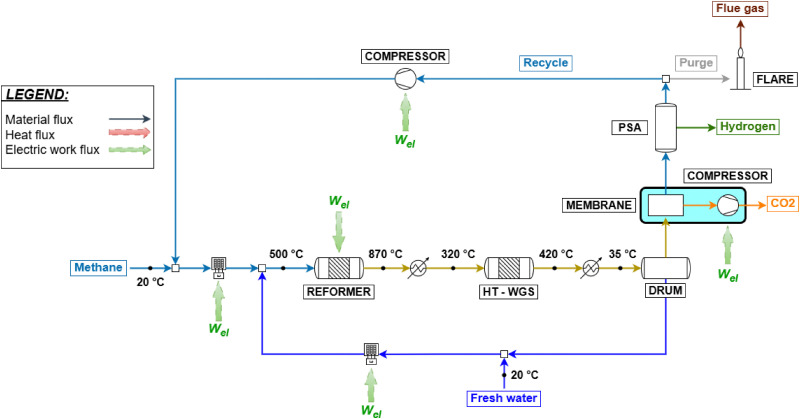
e-SMR plant flow sheet; equipment additions due to CCS implementation are highlighted.

The last process analyzed in this work is the electrified steam methane reforming (e-SMR). The process scheme is reported in Fig. 6. Apart from the consequences of the reduced scale of the process (as for biogas reforming), the main difference with the benchmark natural gas reforming consists in the replacement of the combustion-driven heat supply with the use of electrical resistances and of process-to-process heat exchangers with electric devices. Another possible technology for reforming electrification consists in the use of an induction-based heating system, which is not discussed in this work due to the low technology readiness level (TRL) of the process.^28^ The size of the e-SMR plant is selected to obtain an electrical duty for the reformer of around 1 MW_el_, which, according to the study by From *et al.*,^27^ is a suitable size to optimize the energy efficiency of the reactor. This allows a direct comparison with the biogas reforming case with an average electrolyzer stack,^71^ enabling consistent performance comparisons among the different technologies. Due to the reduced scale of the system, no HDS unit is considered, which leads to the need to use a sulfur-free feed (grid-derived methane), with a volumetric flow equal to 250 Nm^3^ h^−1^. The whole feed stream is delivered to the reactor, whose electric consumption is modeled according to the scale-up model by From *et al.*^27^ This predicts a 99% electric efficiency for a 1 MW reformer. This is due to the main energy loss mechanism in the e-SMR, which is the heat dissipation to the surroundings through the surface area of the reactor. Consequently, as the volume of the e-SMR increases with scale up, the relative surface area and heat loss become lower. The electric efficiency is defined as12
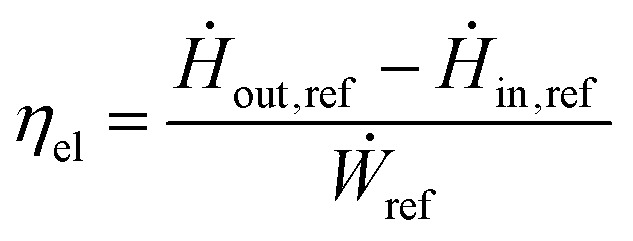
where *Ḣ*_out,ref_, *Ḣ*_in,ref_, and *Ẇ*_ref_ are the enthalpy flow of the reformer outlet, the enthalpy flow of input stream, and the electric duty to supply to the reformer to reach equilibrium at the reactor outlet temperature, respectively. The syngas produced follows the same downstream processing sequence as in the biogas PSWA plant. Similarly, the CCS unit, when included, employs selective membranes. The same is applied to the PSA unit, which achieves a hydrogen recovery efficiency of 90%. Due to the impossibility of using tail gas as a fuel (the heating system is fully electrical), the stream is partially purged and subsequently compressed from the desorption pressure (1.0 bar) to the operating plant pressure and recycled to the inlet methane feed. The purge is flared to convert the remaining traces of CO and CH_4_ to CO_2_, and the purge ratio (*β*), which is defined as follows, is chosen by sensitivity analysis:13
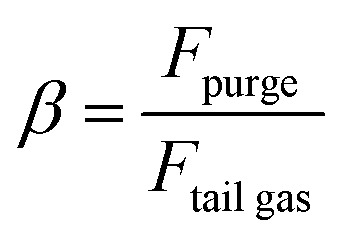
where *F*_purge_ and *F*_tail gas_ are the molar flowrate of purge and tail gas of the PSA streams, respectively. A sensitivity analysis assesses the influence of process parameters on plant efficiency metrics to optimize their values. In this specific case, the volumetric energy cost of hydrogen is assessed:14
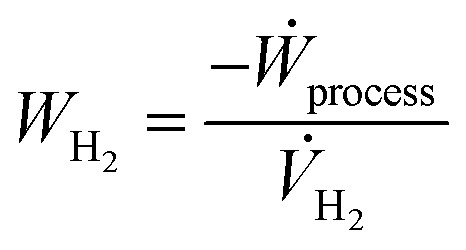
where *Ẇ*_process_ and *V̇*_H_2__ represent the electric power produced by the e-SMR process (kW) and the volumetric flow rate of the hydrogen produced (Nm^3^ h^−1^), respectively. The negative sign is due to the definition of the variables. Based on the analysis, the purge ratio is set to 0.2 for the base plant and 0.01 for the CCS-equipped plant. This choice reflects the need to remove as much inert CO_2_ as possible, thereby reducing the heating duty required for the reformer feed stream. The selected values aim to optimize the hydrogen production cost while avoiding excessively low plant productivity, as illustrated in Fig. A.10. A similar analysis is performed for the choice of plant operating pressure and for the reformer outlet temperature. Pressure is investigated in the 5–20 bar range, while the temperature is between 750 and 1000 °C. Both ranges are used by the e-SMR pilot plant investigation performed by From *et al.*^27^ The specific energy trend exhibits a parabolic behavior, with optimal points emerging for each configuration and pressure level, as shown in Fig. A.11 and A.12. A pressure of 10 bar is selected as a compromise between the minimization of the specific hydrogen cost and the reduction of the operating volumes, as discussed in previous sections. This selection results in optimal reformer outlet temperatures of 780 °C and 870 °C for the base plant configuration and the one with CCS implementation, respectively. The main process variables are summarized in Table A.7.

### 2.5 Benchmark low-carbon technologies for hydrogen production

In addition to the SMR and ATR process simulations, alkaline water electrolysis (AWE) and methane pyrolysis are investigated as a benchmark for small-scale, low-carbon hydrogen production. The choice of alkaline water electrolysis is due to its high technology readiness level (TRL), which defines it as the most mature commercial technology for water electrolysis.^72^ Hydrogen production takes place in modular electrolyzers, consisting of an anode and a cathode, separated by a diaphragm. The following electrochemical reactions occur in the process:

15 Anode: 4OH^−^ → 2H_2_O + O_2_ + 4e^−^

16 Cathode: 2H_2_O + 2e^−^ → H_2_ + 2OH^−^

17 Overall: 2H_2_O → 2H_2_ + O_2_

Despite the technological interest in this route due to the low carbon emissions of the process, water electrolysis shows limited energy efficiencies, ranging between 60 and 70%.^73^ This results in a considerable energy cost (around 54 kWh kg_H_2__^−1^ for AWE^13^). Moreover, a significant amount of water is consumed by the process: assuming that the global demand for hydrogen is met by water electrolysis, a quantity close to 1.3% of the total current water consumption due to energy production would be required.^74^ The limitations related to energy consumption, process efficiency, and water availability have changed the focus of low-carbon hydrogen production toward alternative pathways. Due to its lower reaction enthalpy compared to methane steam reforming (eqn (9)), methane pyrolysis shows a reduced energy intensity. As energy requirements ranging between approximately 10 and 30 kWh kg_H_2__^−1^ are feasible when plasma-based technologies are employed,^75^ lower consumptions than alkaline water electrolysis (AWE) are demonstrated,18
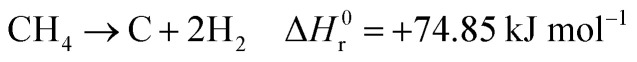


Different technologies for methane pyrolysis are present, such as thermal plasma pyrolysis, distributed electrified heating technology, microwave-driven pyrolysis and molten salt-driven methane pyrolysis.^76^ For the scope of this work, electrified methane pyrolysis was employed as an additional comparison point as it uses electricity to produce H_2_ with an alternative path to the e-SMR process. AWE and electrified methane pyrolysis are not modeled in detail in this work because the related process metrics are directly obtained from other works.^13,76^

### 2.6 Performance metrics for technology evaluation

The final aim of this work is to compare the different technologies discussed and simulated from technical, environmental, and economic perspectives. To this end, appropriate process metrics are developed to quantify the performance of the plants under study.

#### 2.6.1 Technical assessment

Technical analysis is fundamental to evaluate performance from a design point of view and to compare large-scale benchmark hydrogen technologies with emerging small-scale ones. The main goal of this section is to assess the productivity and efficiency of each process, highlighting how the diiferent processes operate and how effectively they convert different feedstock into hydrogen. To this end, two different efficiencies are defined: LHV-based plant efficiency and energy plant efficiency. The former is used as a benchmark to compare the performance of SMR and ATR plants, with and without CCS, with the results reported by Antonini *et al.* (2020),^36^ whose study closely resembles the current work section on gray and blue hydrogen production through natural gas reforming. Moreover, parametric analysis discussed in Section 2.2 aims at optimizing this metric for the definition of suitable process parameters. The LHV-based plant efficiency is defined as follows:19
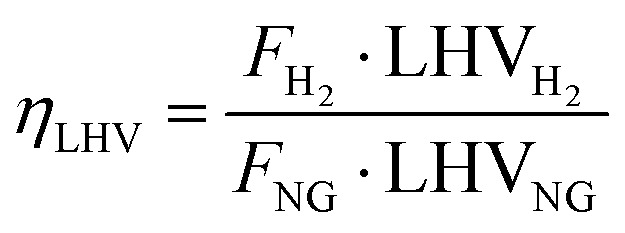
where *F*_H_2__ and *F*_NG_ are product hydrogen from plant simulation and natural gas molar flow rates, while LHV_H_2__ and LHV_NG_ are lower heating values of hydrogen and natural gas, equal to 240 MJ kmol^−1^ and 832.8 MJ kmol^−1^, respectively. With natural gas being a mixture of several chemical species, LHV_NG_ is assessed as follows:20
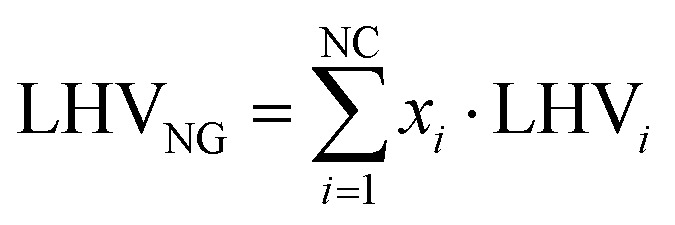
with NC being the number of compounds in the mixture (assumed to be 5, based on Table 2), *x*_*i*_ the molar fraction of each species in the stream and LHV_*i*_ the respective lower heating value on a molar basis. This metric is useful for the natural gas reforming process because the inlet and outlet streams represent the vast majority of the energetic input and output to the plant. The same does not apply to the small scale technologies, as the replacement of process heat recovery with electric devices results in a larger impact of external electric sources on the plant performances. To better compare the latter with natural gas reforming, a new energy efficiency metric is introduced, which accounts for the impact of external energy inputs:21
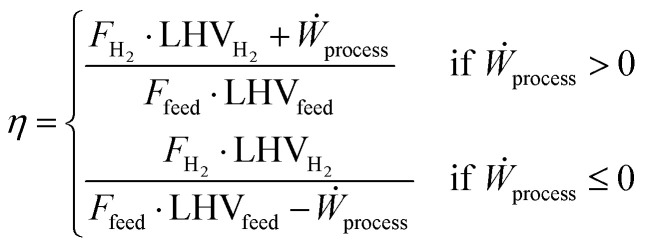
where *Ẇ*_process_ is the electrical work produced by the process, measured in MW, whilst the molar streams and LHVs of input (*F*_feed_, LHV_feed_) and output (*F*_H_2__, LHV_H_2__) are reported in kmol s^−1^ and MJ kmol^−1^, respectively. Since external heat in small-scale processes is assumed to be entirely supplied *via* electricity, the contribution of thermal heating is not considered by this metric. In addition, the generation of electricity from steam turbines in natural gas reforming processes requires the definition of two distinct parameters, depending on whether the plant consumes or produces electric energy.

**Table 2:** Composition of natural gas in molar fractions

CH_4_	C_2_H_6_	C_3_H_8_	CO_2_	N_2_	H_2_S	COS
0.89	0.07	0.01	0.02	0.01	5 ppm	2 ppm

#### 2.6.2 Environmental assessment

The environmental comparison of the processes is carried out through the assessment of the so-called carbon footprint (CFP). This parameter quantifies the specific CO_2_ emissions associated with the process, normalized to the amount of hydrogen produced, and is expressed in kg_CO_2__ kg_H_2__^−1^. This metric includes emissions due to both the productive process (*e.g.*, fuel combustion, CO conversion to CO_2_ through WGS) and the use of grid-derived electricity. For the latter, the carbon footprint of the national electricity grid is assumed as the 2024 averages, based on the reference database.^77^ The previous assumptions lead to the following definition of CFP:22
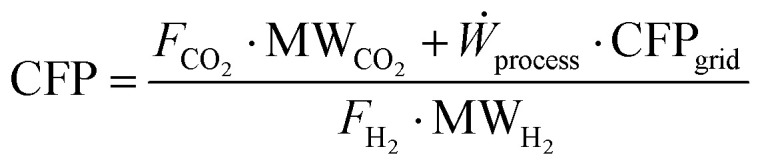
where *F*_CO_2__ and *F*_H_2__ are the molar flowrates of process-based CO_2_ emissions and product hydrogen, and CFP_grid_ is the carbon footprint of the electric grid in kg_CO_2__ kWh^−1^, while MW_CO_2__ and MW_H_2__ are carbon dioxide and hydrogen molar weights, expressed in kg kmol^−1^. In the natural gas reforming process, the carbon dioxide content in the feed is excluded from the overall carbon emission flow rate. In biogas reforming, the only source of fossil carbon emissions is the electricity supply, as biogas-derived CO_2_ is assumed to be biogenic and therefore is not considered to have an environmental impact in this evaluation. Finally, in electrified steam methane reforming, carbon emissions arise from both the flare and the electricity supply.

#### 2.6.3 Economic analysis

The last assessment consists of the evaluation of the appropriate metrics for the economic characterization of the different technologies. Among these, FLOX® is excluded from this study, since conducting an economic analysis on such a smaller scale than the other processes would not be meaningful. As the target of this study is Europe, euro (€) is chosen as the currency. The first step in the assessment is represented by the estimation of the annual cost of fuel consumption as a result of the use both as a feed and as a heating source. The feed-specific cost (*C*_feed,LHV_) is based on both the literature and open-source databases and is assumed to be equal to that reported in Table 3.

**Table 3:** Specific fuel costs

*C*_feed,LHV_	Value	Ref.
Natural gas cost	32 € per MWh	78
Biogas cost	60 € per MWh	79
Methane cost	32 € per MWh	78

The cost of biogas is hypothesized to be equal to the average value reported for biogas digesters in Europe, while the cost of natural gas is taken as the rough average price in the 2023–2025 time series. The reported costs are converted to € kmol^−1^ using their respective molar LHV values (800 MJ kmol^−1^ for methane, 832.8 MJ kmol^−1^ for natural gas, and 440 MJ kmol^−1^ for biogas). As further assessments require the calculation of annualized costs, plants are considered to be operating for 90% of the available yearly hours (7884 h per year), named in further sections as *N*_hours_. This leads to the definition of the yearly fuel cost from feed specific cost (*C*_feed,LHV_), LHV (LHV_feed_) and molar flowrate (*F*_feed_):

23 *C*_fuel_ = *F*_feed_·LHV_feed_·*C*_feed,LHV_·*N*_hours_ (€ per year)

where the reference feed cost is converted from € per MWh to € per MJ. To provide financial incentives for the implementation of carbon-reducing technologies, the EU has proposed carbon credits, which reward companies with a certain amount of money for each ton of CO_2_ emissions avoided. This enables a deeper analysis of the economic feasibility of CCS adoption. It was considered that carbon credits could be applied to this case, as the application of CCS to H_2_ production is a typical case of CO_2_ emission reduction for chemical plants/oil refineries. This assumption may be verified on each specific cases, according to the legal framework of the H_2_ production plant. In this work, carbon credits (CO_2credits_) are valued at 80 € per t_CO_2__, according to the average value reported in a reference database.^80^ The yearly carbon credits (CO_2profit_) are assessed as follows:

24 CO_2profit_ = *F*_CO_2__·MW_CO_2__·CO_2credits_·*N*_hours_ (€ per year)

where *F*_CO_2__ and MW_CO_2__ are the molar flowrate and the molar weight of the carbon dioxide captured. The capital cost (CAPEX) of the plants is assessed in accordance with the literature and arises from the estimation of the total costs due to the erection and installation of the various equipment in the processes. The bare erected cost (BEC), which consists of the cost related to the purchase of the devices, is initially evaluated by adopting a cost-scaling correlation defined as follows:25
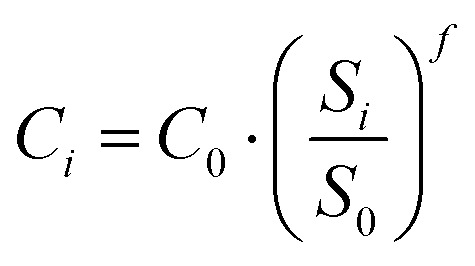
where *C*_*i*_ is the cost of the given equipment and *S*_*i*_ is its capacity, whilst *C*_0_ and *S*_0_ are the reference cost and capacity of the same equipment, respectively. Finally, *f* is the so-called scaling factor. The reference values for this work are assumed according to the study by Spallina *et al.*,^38^ except for the case of membrane-based carbon capture technology, which is based on the work of Lin *et al.*^63^

The costs of the WGS section include both HT and LT reactors, and thus the related BEC is divided by two in case LT-WGS is not foreseen by the layout. A similar approach is applied to estimate the bare erected cost of compressors (BEC_CPR_) based on their electrical requirements (*W*), using the correlations proposed by Ulrich *et al.*^40^

26 BEC_CPR_ = 1 × 10^−5^*W*^3^ − 0.1074*W*^2^ + 789.44*W* + 4116.3

The estimated cost of heat exchangers (BEC_HE_) is derived from the results reported in ref. 39, applying the same cost ratio, equal to 0.31, between these components and the bare erection cost associated with reactors, compressors, turbines and PSA (BEC_vessels_). This approach represents a conservative approximation when applied to technologies different from natural gas reforming, as distinct electric devices are used in e-SMR and biogas reforming systems,

27 BEC_HE_ = 0.31·(BEC_vessels_ + BEC_CPR_)

To account for the price variation of the instrumentation along time and use updated cost values, CEPCI coefficients are exploited:28
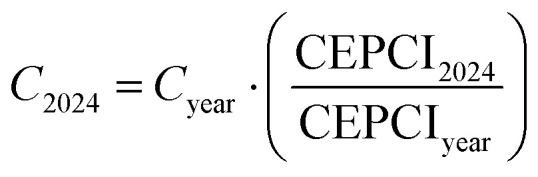
this calculation allows assessing the updated equipment cost to June 2024 (*C*_2024_) by correcting the value evaluated based on Table 4 for a given year (*C*_year_). The CEPCI coefficients' year is related to the given subscript, and their value is based on an open-source database.^84^ Given that BEC represents the sum of the bare erected cost for all equipment, CAPEX is calculated as follows based on the literature.^85^ TIC represents the total installation cost of the equipment, which arises from BEC,

29 TIC = 0.8·BEC

**Table 4:** Reference data and scaling factors for the main process equipment

Equipment	Scaling parameter	*S*_0_	*C*_0_ (M€)	*f*	Cost year	Ref.
HDS	Plant input (MW_LHV_)	413.8	0.66	0.67	2011	81
WGS reactors	Plant input (MW_LHV_)	1246.06	9.54	0.67	2007	82
Reformer + pre-reformer	Plant input (MW_LHV_)	1246.06	42.51	0.75	2007	82
Pre-reformer	Plant input (MW_LHV_)	1800	17.50	0.75	2005	83
PSA unit	Inlet flow rate (kmol h^−1^)	17.069	27.96	0.60	2007	82
Steam turbine	ST gross power (MW)	200	33.70	0.67	2007	81
Amine unit (MDEA)	CO_2_ separated (kg s^−1^)	68.2	46.14	0.80	2011	82
Membrane CCS	CO_2_ separated (kg s^−1^)	9.86	0.875	0.80	2015	63

TIC is added to BEC to estimate the total direct plant cost (TDPC):

30 TDPC = TIC + BEC

Indirect costs (IC) are assumed to be equal to 14% of TDPC:

31 IC = 0.14·TDPC

IC is added to TDPC to obtain engineering, procurement, and construction cost (EPC):

32 EPC = IC + TDPC

The latter is then used to estimate the so-called contingencies (*C*), which account for the unpredictable expenses that may arise during plant construction:

33 *C* = 0.1·EPC

EPC and contigencies are merged to assess the total overnight cost (TOC):

34 TOC = *C* + EPC

Maintenance costs (*M*) are estimated from TOC:

35 *M* = 0.025·TOC

The insurance cost (*I*) is calculated analogously to *M*:

36 *I* = 0.02·TOC

CAPEX is defined as the sum of TOC, *M*, and *I*, distributed with a fixed recovery factor over the expected plant lifetime (*N*_years_), which is assumed to be 25 years, based on the study by Collodi *et al.*,^30^37
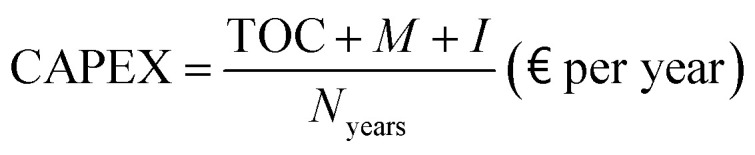


In addition to the annual capital cost of the plant, the contribution of the operating costs (OPEX) required to run the process is investigated. OPEX is assumed to include costs for water utility (for both steam production and cooling systems), catalyst, labour, and CO_2_ transport and storage. Although electricity cost is usually included within this parameter, the present work reports a separate dynamic analysis for this component. The process water is intended for steam generation and corresponds to the make-up stream required to achieve the fixed S/C ratios determined from previous plant simulations. In contrast, the cooling water flow rate is estimated according to the specific process under examination. For the SMR and ATR configurations, the value is obtained from the data reported in ref. 39, appropriately scaled to reflect the different feedstock flow rates considered in the two studies. This adjustment accounts for the exclusion of cooling water in the present SMR and ATR configurations, due to the modeling simplifications in the equipment design. For biogas and e-SMR cases, the cooling water requirements are estimated from the respective cooling duties resulting from simulations, assuming that cooling water undergoes a 30 °C temperature increase. In addition, the water requirements of the PSWA unit are added to the overall demand of process water discussed above. A similar approach to that for fuel cost is used to assess the yearly cost of water utilities (*C*_water_):

38 *C*_water_ = (*V̇*_w,process_·*C*_w,process_ + *V̇*_cw_·*C*_cw_)·*N*_hours_ (€ per year)

where *V̇*_w,process_, *V̇*_cw_, *C*_w,process_, and *C*_cw_ are the volumetric flow rates of process and cooling water and the respective specific costs. Catalyst cost assessment accounts for the requirements of HDS, WGS and reforming reactors and is based on the literature. The required catalyst volumes (*V*_*i*_) are derived from ref. 39 and are expressed on a molar basis, allowing the values to be scaled proportionally to the process feedstock flow rate (*F*_feed_). The yearly catalyst cost (*C*_cat_) is estimated based on ref. 38 and adjusted according to the expected catalyst lifetime (*L*), 5 years,39
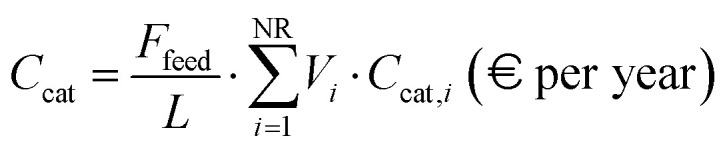
where *C*_cat,*i*_ represents the volumetric cost of each of the NR catalyst types considered. The yearly labour cost (*C*_labour_) is selected to be equal to the one reported in ref. 29 for SMR plants (*C*_0,labour_) and scaled according to adopted (*F*_feed_) and reference feed flow (*F*_0,feed_), using a factor *f* equal to 0.75,40
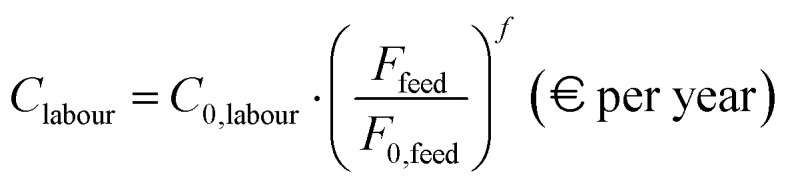


A similar approach is used for the yearly cost related to CO_2_ transport and storage into pressurized vessels (*C*_CO_2__), which is selected in accordance with the IEAGHG report, 2017,^29^ and scaled with a factor *f* of 0.8,41
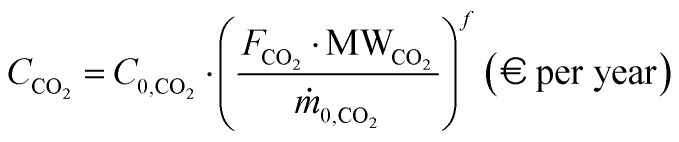
where *C*_0,CO_2__ is the reference-based cost for CO_2_ storage, and *m*_0,CO_2__ is the reference-based captured CO_2_ mass flowrate, while *F*_CO_2__ and MW_CO_2__ are the captured CO_2_ molar flowrate and molar weight. The values adopted in this section are summarized here (Table 5).

**Table 5:** Reference values for water, labour and catalyst costs

Item	Value
SMR-ATR cooling water	0.56 m^3^ kmol_feed_^−1^
*C*_w,process_	2 € per m^3^
*C*_cw_	0.35 € per m^3^
*V*_HDS_	1.89 ft^3^ (kmol_feed_^−1^ h^−1^)
*V*_Ref_	0.28 m^3^ (kmol_feed_^−1^ h^−1^)
*V*_WGS_	0.19 m^3^ (kmol_feed_^−1^ h^−1^)
*C*_HDS_	319 € per ft^3^
*C*_Ref_	10 000 € per m^3^
*C*_WGS_	2800 € per m^3^
*C*_0,labour_	2.3 M€ per year
*C*_0,CO_2__	3.7 M€ per year

It is worth specifying that, although LT and HT reactors employ different types of catalysts, their cost is assumed to be the same. Plant operation costs are finally evaluated, accounting for water utilities (*C*_water_), carbon transport and storage cost (*C*_CO_2__), catalyst and labour cost (*C*_cat_, *C*_labour_):

42 OPEX = *C*_water_ + *C*_CO_2__ + *C*_cat_ + *C*_labour_ (€ per year)

The assessment of electricity cost is based on real, nation-specific, and time-resolved electricity prices obtained from the EPEXSPOT database.^34^ Specifically, day-ahead auction prices are used for the analysis. The countries considered in this study are Germany (DE), France (FR), and Switzerland (CH). Two different representations of electricity prices are employed: the average value for each country in the January 2023–October 2025 period (*C*_el,avg_), which allows for a static but realistic assessment, and the sorted hourly prices for each scenario, allowing for a dynamic, time-resolved evaluation. The static yearly electricity cost is estimated as follows, assuming 7884 yearly operating hours (*N*_hours_):

43 *C*_el_ = −*Ẇ*_process_·*C*_el,avg_·*N*_hours_ (€ per year)

the negative sign is due to the definition of *Ẇ*_process_, as its value is set as negative when electricity is supplied externally. The exploited average electricity prices, obtained from proper data analysis, are reported in Table 6.

**Table 6:** Average electricity prices in Jan 2023–Oct 2025 time series^34^

Country	*C*_el,avg_ (€ per MWh)	CFP (kg_CO_2__ kWh^−1^)
France	72.84	33.45
Germany	91.25	333.78
Switzerland	93.86	41.07
Italy	116.29	274

The parameters described above are ultimately used for the calculation of the levelized cost of hydrogen (LCOH), which is the main metric used for the economical comparison of the various technologies. This accounts for the yearly costs related to feed supply (*C*_fuel_), electricity (*C*_el_), carbon credits (CO_2profit_), and capital and plant operation (CAPEX, OPEX),44
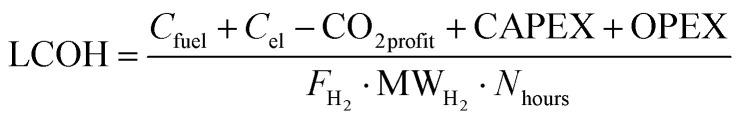
where the denominator indicates the yearly massive production of hydrogen for the process under examination. The LCOH variable is measured in € per kg_H_2__. However, the price of electricity shows huge variations between days and months of the year, as shown in Fig. 15, A.13–A.15. Given the strong dependence of e-SMR on electricity consumption, along with its quick start-up procedure, the electricity price time series is analyzed to determine the threshold above which reactor shutdown is economically preferable. To this purpose, electricity prices are sorted for each scenario, and the dynamic LCOH corresponding to the cumulative operation up to each considered electricity price is evaluated. Focusing on the January 2023–October 2025 time span, the annualized fixed costs of the plant, comprising CAPEX and time-independent cost variables in the previous OPEX definition (*C*_cat_, *C*_labour_, and *C*_CO_2__), as evaluated within the static LCOH framework, are employed to compute the total fixed costs over the number of hours in the time series (*n*_series_). A 90% plant operation is assumed, consistent with the static LCOH assessment, corresponding to 7884 annual operating hours,45
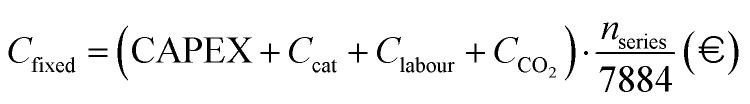


In addition to fixed costs, operating costs associated with fuel, water, and electricity supply to the process (*h*_cost,*i*_), as well as revenues from CO_2_ credits (*h*_rev,*i*_) and hydrogen production volumes (*h*_prod,*i*_), are evaluated for each *i*-th ordered operating hour. It should be noted that the *i*-th hour includes cumulative values accounting for all preceding hours in addition to the contribution of the hour itself. The variation in the *n*-th hourly electricity price (*C*_el,*n*_) implies a more complex definition. Here, *i* denotes the position of the selected hour within the overall time interval,46
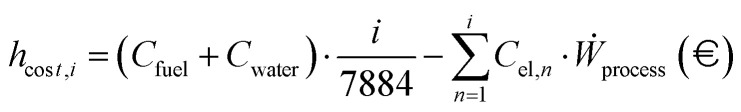
47
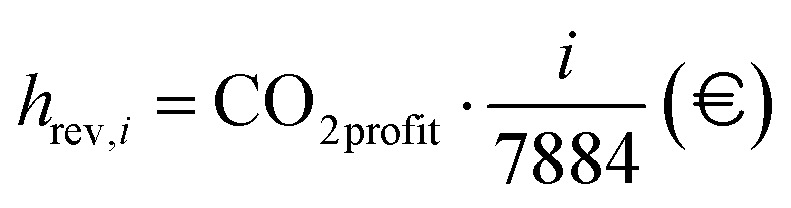
48
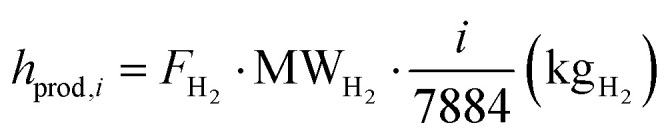


The dynamic LCOH for the *i*-th hour is finally calculated based on the reported cost variables. Aside from a different use of the latter, the identity is analogous to eqn (44) under a mathematical standpoint,49
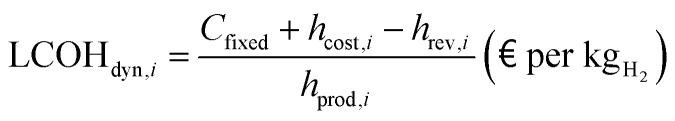


#### 2.6.4 Metrics evaluation for benchmark low-carbon hydrogen production

In addition to the comparison among SMR, ATR, biogas reforming, and e-SMR, a further analysis is conducted to provide a preliminary assessment of environmental and economic metrics for hydrogen production *via* alkaline water electrolysis (AWE) and methane pyrolysis. For methane pyrolysis, carbon footprint (CFP_pyrolysis_) is estimated from other works,^76^ showcasing an energy consumption (*W*_pyrolysis_) of 9.7 kWh kg_H_2__^−1^. Electricity supply is modeled as attained through national grid (CFP_grid_):

50 CFP_pyrolysis_ = *W*_pyrolysis_·CFP_grid_

Analogously, for AWE technology, carbon footprint (CFP_AWE_) is estimated based on the reported energy consumption (*W*_AWE_), which is equal to 54 kWh kg_H_2__^−1^,^13^

51 CFP_AWE_ = *W*_AWE_·CFP_grid_

Besides technical metrics assessment, the literature highlights hydrogen costs from methane pyrolysis ranging from a best-case 5.4 (France) to a worst-case 6.6 € per kg_H_2__ (Germany and Switzerland), depending on electricity price.^76^ Under the assumption of a constant CAPEX, the LCOH from AWE is interpolated as a linear function of national electricity price (*C*_el,avg_), with values of 3.2 (LCOH_AWE,40_) and 4.6 € per kg_H_2__ (LCOH_AWE,60_) corresponding to electricity prices of 40 and 60 € per MWh, respectively,^13^52
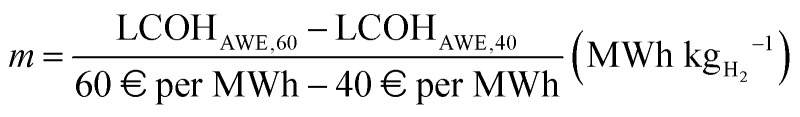

53 *q* = LCOH_AWE,60_ − *m*·60 € per MWh (€ per kg_H_2__)

54 LCOH_AWE_ = *m*·*C*_el,avg_ + *q* (€ per kg_H_2__)

## 3 Results

A detailed analysis of the metrics resulting from the simulation of the plants under investigation is reported here. The supporting data are presented in Tables A.7 and A.10 in the SI.

### 3.1 Technical metrics

**Fig. 7:**
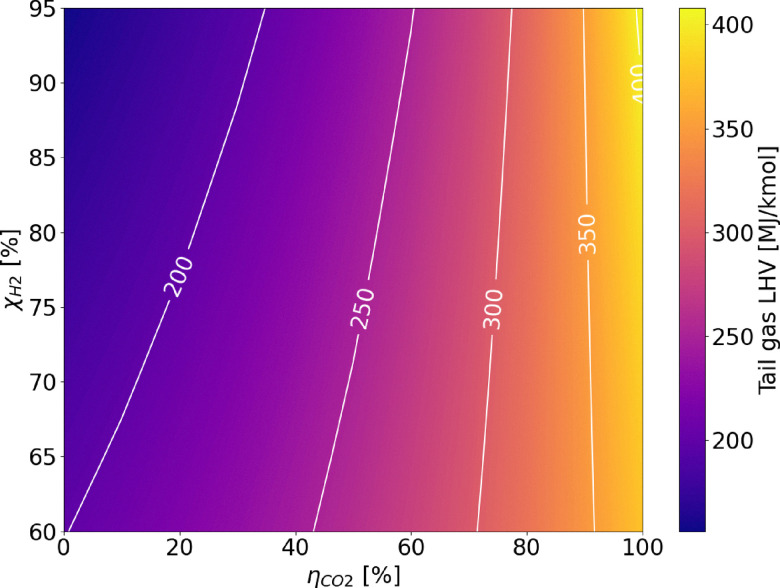
Analysis of tail gas LHV as a function of CO conversion *via* the WGS reaction (*χ*_CO_) and CO_2_ unit capture efficiency (*η*_CO_2__).

**Fig. 8:**
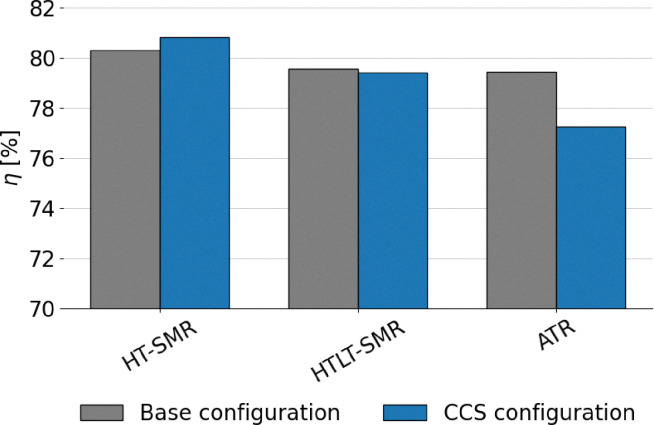
Comparison between SMR and ATR in terms of energy plant efficiency.

The extensive process integration of benchmark SMR and ATR technologies, made possible by the large scale of the processes, results in high LHV-based plant efficiency, ranging between 76 and 80% depending on the layout. The assumptions made are validated against the results in the study by Antonini *et al.*^36^ The comparison with the reference values is shown in the SI (Fig. A.1) and reveals very similar metrics and comparable carbon footprints for the plants under examination. The addition of carbon capture is always beneficial for the process efficiency parameter. This effect occurs because CO_2_ is inert during combustion, and hence its removal increases the specific lower heating value (LHV) of the tail gas. Consequently, the natural gas demand to the burner decreases, allowing a greater fraction of the feed to be directed to the reactor. This increases the overall productivity of the process, as shown in Fig. 7 and A.16. In contrast, the integration of the LT-WGS reactor into the process layout leads to a higher CO-to-CO_2_ conversion (around 90%), which decreases the LHV of the tail gas and, as a consequence, results in lower plant efficiency compared to HT-SMR. This effect is not balanced by the hydrogen production resulting from the enhanced WGS reaction. Apart from improvements in terms of efficiency and productivity, the addition of carbon capture enables a reduction of roughly 60–65% in the carbon footprint for SMR schemes compared to gray hydrogen, which increases to 85% for the ATR process. This is because, unlike SMR, the ATR layout allows the capture of CO_2_ produced from the natural gas that is burned to sustain the auto-thermal operation of the reactor. Furthermore, the higher temperatures and complete oxygen conversion inside the reactor lead to higher natural gas conversions compared to SMR and thus lower hydrocarbon traces in the tail gas. As a result, CO_2_ emissions from the combustion of the latter are reduced compared to SMR plants with CCS. In addition to the efficiency (*η*_LHV_) analysis, the balance of electricity produced and consumed by the plants levels the advantages of CCS configurations over the conventional layout, except for the HT-WGS case (which benefits from the absence of LT-WGS in terms of efficiency), as shown in Fig. 8. This trend is observed as a consequence of the lower net power generation in CCS plants (Table A.7), mainly caused by the lower flue gas volume, which results in reduced excess steam production (Fig. A.17). Moreover, a significant fraction of the generated power is required for CO_2_ compression. This effect is even more pronounced in the ATR configuration, which employs an external air separation unit for pure oxygen production, resulting in a further increase in the overall power demand, resulting in a 77.3% efficiency in case CCS is employed. In addition to that, in the ATR process, the CO_2_ removal performs better compared to SMR, but it is also more energy-intensive, thus requiring additional purchase of electricity to perform the compressions. Regarding power production, the introduction of the LT stage boosts the electrical output. This results from the higher methane content in the overall stream derived from the mixing of fuel and tail gas (19 mol% compared to 17 mol% in the HT-SMR case), which is due to the lower energy content of the tail gas, as mentioned above. Although the total flow of fuel is slightly lower, the combustion of methane requires a higher air flow than the other components due to stoichiometry (eqn (A.55)–(A.57)), resulting in greater flue gas production, which increases the available process heat and the consequent excess steam generation through their cooling in the heat recovery section. The best performing configuration is represented by HT-SMR with CCS implementation with 80.8% efficiency, while the addition of LT-WGS is shown to be not beneficial for hydrogen production within the described processes.

**Fig. 9:**
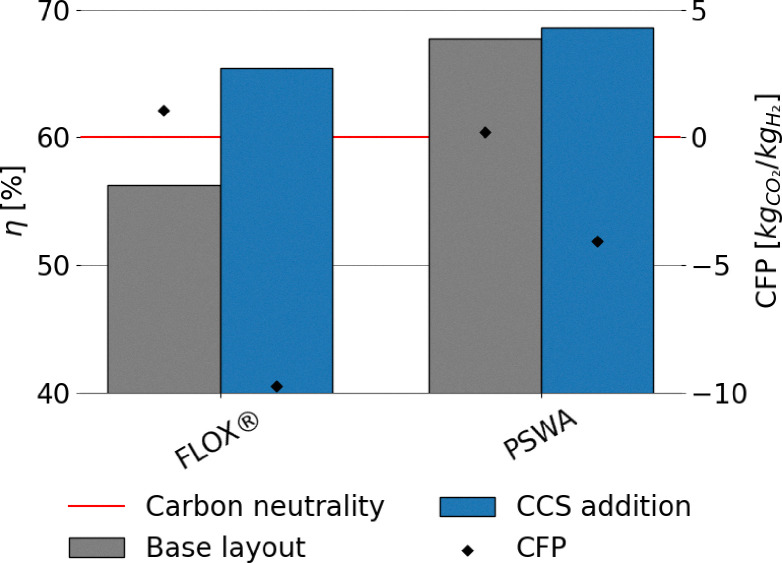
Energy efficiency and fossil carbon footprint of biogas conversion to hydrogen using Italian grid as the source of electricity.

**Fig. 10:**
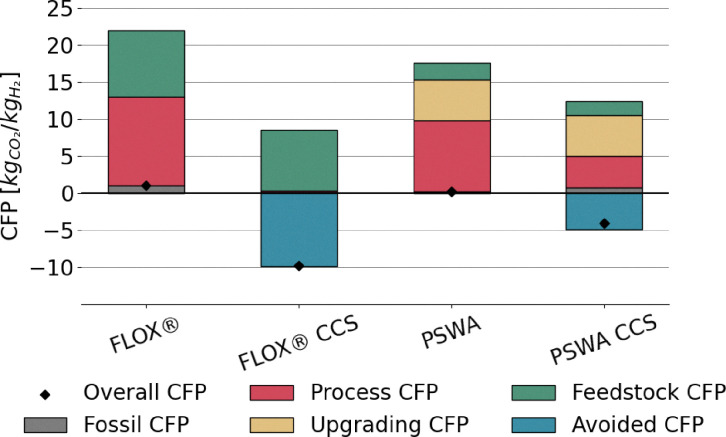
Carbon footprint derived from biogas conversion to hydrogen using Italian grid as the source of electricity. The feedstock CFP is the CO_2_ from biogas that is emitted. The process CFP is the CO_2_ formed in the process (*e.g.*, by combustion), but of biogenic source. The upgrading CFP is the CO_2_ removed by scrubbing for biogas upgrading and released in the atmosphere according to this analysis.

Lower energy efficiencies are observed for biogas reforming processes, ranging approximately from 56% to 68% (Fig. 9). This is attributed both to the smaller scale of plant layouts, which limits the effectiveness of process integration, and to the presence of CO_2_. Indeed, CO_2_ burdens the equilibrium conversion of the system. This phenomenon is particularly pronounced when biogas is fed directly to the reformer, as in the case of FLOX®. Additionally, CO_2_ increases the total flow rate, with the consequence that more heat is required to raise the temperature to equilibrium, thus reducing the overall efficiency of the process. This effect is clearly visible in Fig. 9, as the implementation of upgrading *via* PSWA shows significant benefits to the performance of the process, with or without addition of CCS. It should be specified that, despite the smaller size of the process, the compact design of FLOX® offers a better heat recovery compared to PSWA, although its performance is penalized by the use of non-upgraded biogas as feed. In contrast to large scale processes, the addition of CCS leads to improvements in the energy efficiency of the plant. This originates from the absence of direct power generation in the process, which prevents the development of an efficiency gap in favor of conventional layouts, as observed in SMR and ATR configurations (see Fig. 8 and eqn (21)). The replacement of natural gas with biogas results in significant environmental improvements. Due to the biogenic nature of the feedstock, the CO_2_ emissions associated with the conversion of biogas-derived species into carbon dioxide (*e.g.*, CO conversion *via* WGS and CH_4_ oxidation to CO_2_) are excluded from the calculation of the carbon footprint.^18^ A considerable fraction of the total emitted CO_2_ corresponds to the original carbon content of the feedstock, regardless of whether the feed stream is upgraded or not, as shown in Fig. 10. As expected, the addition of CCS strongly mitigates CO_2_ emissions throughout the process. The lower carbon footprint in FLOX® when CCS is implemented is due to the higher CO_2_ molar fraction in the process, arising from the absence of PSWA unit, leading to less efficient hydrogen production, but at the same time to the capture of a higher fraction of carbon dioxide. In fact, when a PSWA is implemented, the biogenic carbon dioxide removed from the feed is released together with water, preventing the need to separate and capture CO_2_. The sole contribution to CFP is represented by fossil-related emissions originated from electricity consumption, which, for a plant located in Italy, (reference nation for this assessment), corresponds to a carbon intensity of 274 kg_CO_2__ MWh^−1^,^77^ which is the average value observed in 2024. In essence, direct reforming is preferred when the target is the establishment of negative emission strategies (high incentive for carbon capture), while reforming after biogas upgrading is the best solution to maximize the H_2_ productivity. In the FLOX® configuration, the addition of CCS further reduces the electricity demand by eliminating vacuum duties of the VPSA system and decreasing the load at the recycle compression. Conversely, in the PSWA case, higher electricity consumption arises from CO_2_ compression and electrical heating for water vaporization, resulting in a higher overall carbon footprint. Biogenic carbon removal is included in the total footprint calculation, resulting in negative emissions in case CCS is deployed, following the assumption that biogenic CO_2_ is climate-neutral.^18–20^ Under this definition, its permanent storage is accounted for as net carbon removal, although biogenic CO_2_ is effectively emitted within the process.

**Fig. 11:**
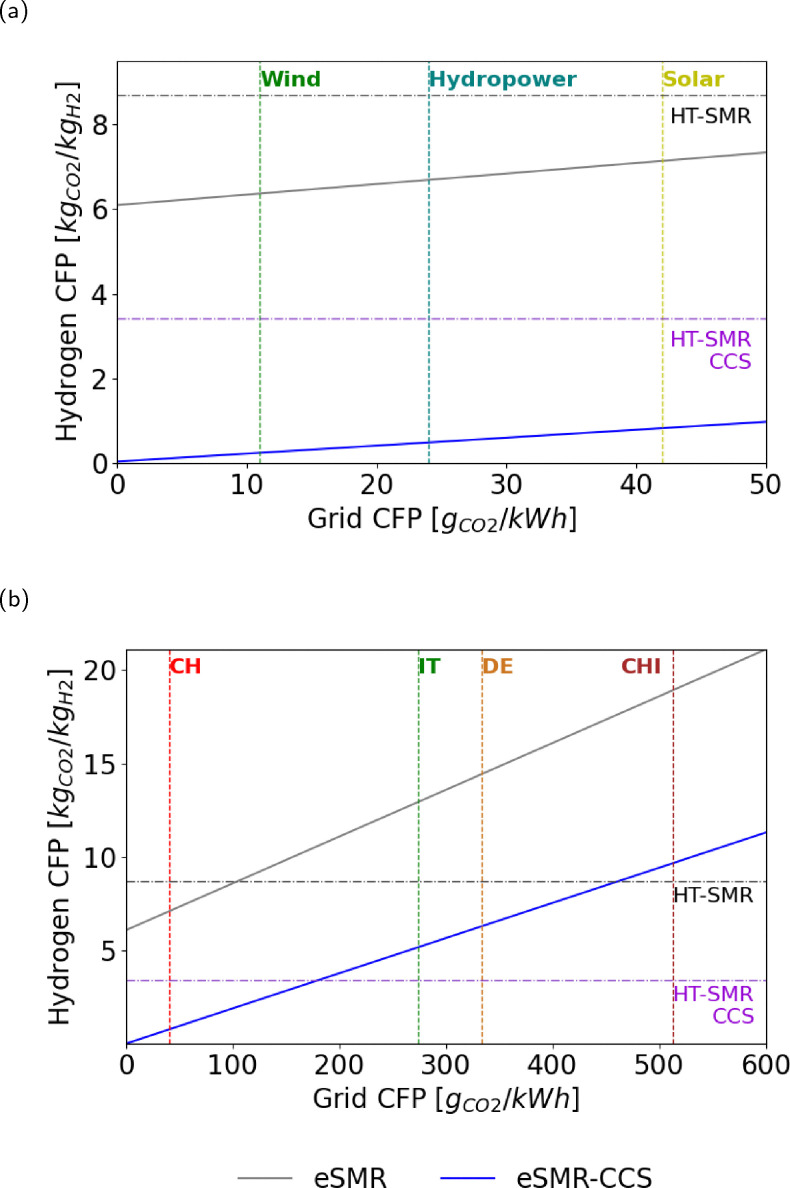
(a) e-SMR carbon footprint (scaled-up version) with and without the CCS unit, based on the electricity grid carbon footprint. Real values are selected for Switzerland (CH), Italy (IT), Germany (DE) and China (CHI); (b) e-SMR carbon footprint (scaled-up version) with and without the CCS unit, by adopting different renewable power sources.

Among the configurations analyzed, the e-SMR process exhibits the strongest dependence on externally supplied electricity, as this replaces the conventional fuel combustion used for process heating. Consequently, the carbon intensity of the electric grid plays a key role in determining the overall emissions of the process (Fig. 11b). Indeed, electricity represents the main source of CO_2_ emissions within the system, with the only additional contribution arising from flaring of the purge gas (Fig. 6). To highlight this dependence, Fig. 11b reports the 2024 average carbon footprint (CFP) values for the Swiss, Italian, German, and Chinese electric grids, allowing for an overview of how different national energy mixes influence the environmental performance of this hydrogen production route. The selected countries were chosen to represent various electricity mix profiles: a low-carbon, net-zero-oriented grid (Switzerland), a fossil-based grid (China), and intermediate cases represented by Italy and Germany. The results indicate that e-SMR without carbon capture does not lead to substantial CO_2_ reduction compared to the conventional HT-SMR benchmark (8.67 kg_CO_2__ kg_H_2__^−1^), as all configurations exceed this reference value except the Swiss case. This suggests that the implementation of e-SMR without CCS is meaningful from an environmental perspective only in contexts where nearly carbon-neutral electricity is available. This is also due to the large purge ratio selected to optimize the overall electrical energy requirements (Fig. A.9), which results in large CO_2_ emissions. However, once carbon capture and storage (CCS) is integrated, a similar linear trend is observed among the cases, but three out of four configurations outperform the gray hydrogen reference. This suggests that e-SMR with membrane-based carbon capture can achieve significant CO_2_ emission reductions even when operated with electricity, which is partially derived from fossil sources. Despite the relevant findings discussed above, the addition of CCS does not allow carbon emissions to remain below the threshold set by HT-SMR with CCS, except in the Swiss case. This indicates that blue hydrogen production from natural gas with carbon capture remains the most effective option for reducing carbon emissions, unless biogenic feedstock or high shares of renewable energy are employed.

**Fig. 12:**
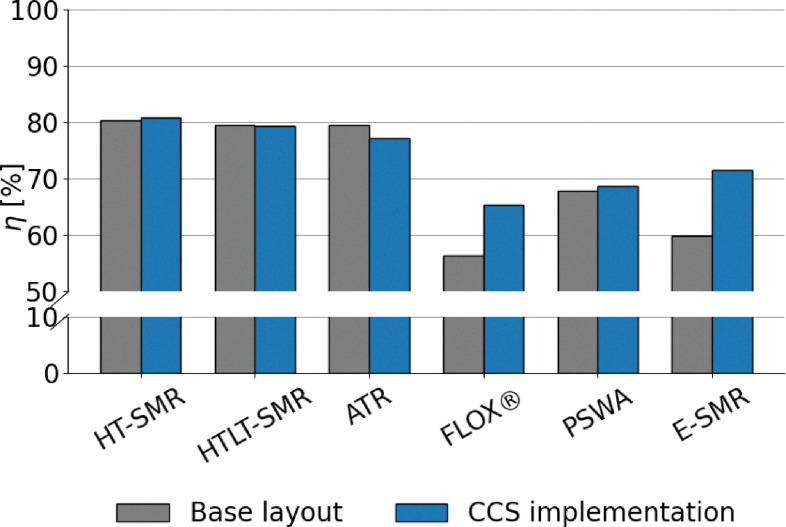
Overall comparison of the energy efficiency of the processes assessed through this work.

As the share of renewable energy is projected to increase continuously in the upcoming years, a complementary analysis is performed assuming that the energy requirements must be fulfilled by different types of clean energy sources, whose indirect CFP values are taken from the literature.^86^ This analysis is of particular interest for assessing the feasibility and potential benefits of future modular e-SMR reactors directly powered by *in situ* renewable energy installations (*e.g.*, photovoltaic panels and wind turbines), similar to what is expected to occur in the near future with water electrolyzers. Fig. 11a highlights the significant improvements in environmental performance obtained through the adoption of fully renewable energy sources for the process energy supply. In both scenarios, the resulting carbon footprints are lower than the threshold defined for HT-SMR processes. The use of 100% renewable energy results in improved performance compared to HT-SMR with CCS, with carbon footprints ranging between 0 and 1 kg_CO_2__ kg_H_2__^−1^, when membrane-based carbon capture is implemented within the e-SMR process. Due to the reduced startup time of the e-SMR reactor (approximately 2 hours, as reported by From *et al.*^27^), and assuming that the national daily energy demand is correctly forecast in advance, coupling this technology with periods of excess renewable energy is highlighted as a promising option for low-carbon hydrogen production in the near future. Fig. A.13–A.15 illustrate the substantial availability of low-cost electricity throughout the year, which generally coincides with periods of excess renewable generation from production facilities. This phenomenon becomes particularly evident when there is a relevant renewable production base load, as observed in the case of Germany (Fig. A.14). For example, the increase in solar generation during summer and at times of low electricity demand leads to a sharp decrease in energy prices. Such conditions create a favorable framework for the type of application discussed above and will be elaborated further in the following section. From an energy performance point of view, a comparable beneficial effect of the addition of CCS (approximately 11%) can be observed in Fig. 12. Unlike previous assessments, this improvement is not due to a more energetic tail gas, as the e-SMR layout adopts a recycle configuration instead. The observed effect can be attributed to the lower energy requirements resulting from the removal of a large fraction of carbon dioxide from the process. Indeed, similar to the case discussed for biogas-based processes, carbon dioxide not only acts as an inert species detrimental to the reaction equilibrium but also increases the volumetric flowrate of the reactor feed. This leads to a sharp reduction in the electricity required to raise the stream temperature to the desired outlet value when CCS is implemented. Despite the absence of process integration, replaced by the use of electrical heaters, e-SMR achieves energy efficiencies comparable to those of small-scale, biogas-derived hydrogen production, reaching up to 71% when CCS is implemented. It should be noted that, different from MDEA-based capture, membrane CCS is assumed to be almost energy-neutral and the sole CO_2_ compression contributes to the electricity consumption. Although not reaching the performance of the benchmark SMR and ATR processes, this novel electrified system exhibits promising efficiency, which could potentially be further improved through process scale-up. In conclusion, the application of carbon capture improves the fuel efficiency of all processes, because it increases the purity of the recycle streams. In particular, the increase in efficiency is large for processes that treat high amounts of CO_2_ due to either the nature of the feedstock (FLOX) or the need of large recycle flow (eSMR). An economic evaluation is then needed to verify the presence of eventual trade-offs with the increased electricity demand of the CCS configurations.

**Fig. 13:**
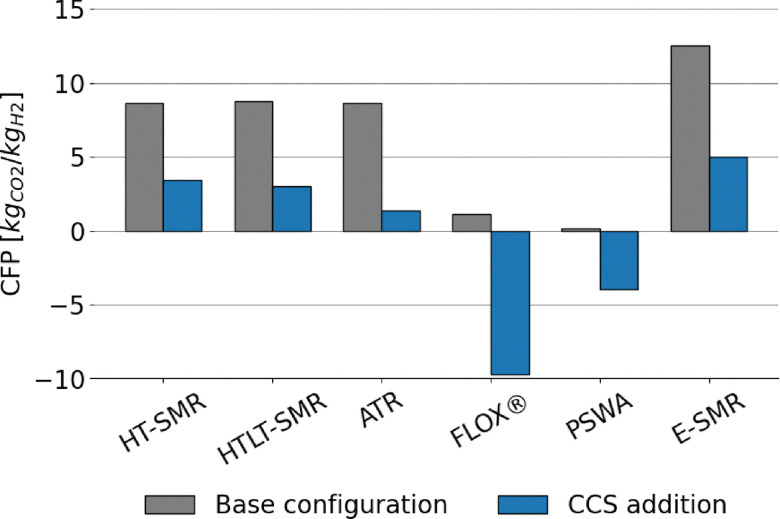
Overall comparison of the carbon footprint of the processes assessed through this work. Electricity supply is assumed to be achieved through the Italian national grid.

A comprehensive comparison of the technologies evaluated is performed in terms of CFP related to hydrogen production, as shown in Fig. 13. As discussed, both e-SMR and, to a lesser extent, biogas CFP are influenced by the grid-related carbon footprint, showing a country-based dependency. For this reason, Italy is selected as the reference nation, while further scenarios are explored in the following section. The introduction of CCS is always beneficial to the reduction of carbon emissions, as expected, resulting in negative carbon footprints in case a biogenic feedstock is employed. The latter represents the best performing technology in terms of environmental footprint among the processes analyzed in this work. However, despite the electrification of the heating system, e-SMR is not competitive from an environmental standpoint compared to conventional processes, when fed with electric energy derived from the Italian grid. A better performance was observed when cleaner energy sources are employed, as in the case of Swiss or French grid, where the process becomes environmentally competitive.

### 3.2 Economic analysis

Before the analysis of scenario-based LCOH, the adopted economical model is validated through a comparison with the results of a previous work, aimed at estimating LCOH of similar plants for natural gas reforming with carbon capture implementation.^29^ The latter analysis is validated exclusively for the HT-SMR and HT-SMR with CCS configurations, since the LCOH of the other technologies is evaluated consistently with the SMR-based cases. This preliminary assessment does not account for CO_2_ credits, in agreement with the assumptions taken in the reference study. Moreover, the IEAGHG report adopts different assumptions in terms of the economic assessment of the plant (for instance, different from this work, the costs for start-up, owner capital, and spare parts are accounted for), and no information is given about the calculation methods for both CAPEX and OPEX. The reference values are extrapolated from the linear correlation between LCOH and fuel cost, assuming a fuel cost of 8.9 € per GJ, consistent with the present work. In Fig. A.2, only slight deviations are observed among the LCOH assessed in the two works, whose presence is likely due to the aforementioned differences in the economical assumptions. In both configurations, CCS implementation translates into a higher hydrogen cost, which reflects the increase in investment costs due to the MDEA process added for carbon capture. Although more expensive, blue hydrogen LCOH is in any case below 2.3 € per kg_H_2__, the threshold market value for hydrogen reported by the reference source.^87^

**Fig. 14:**
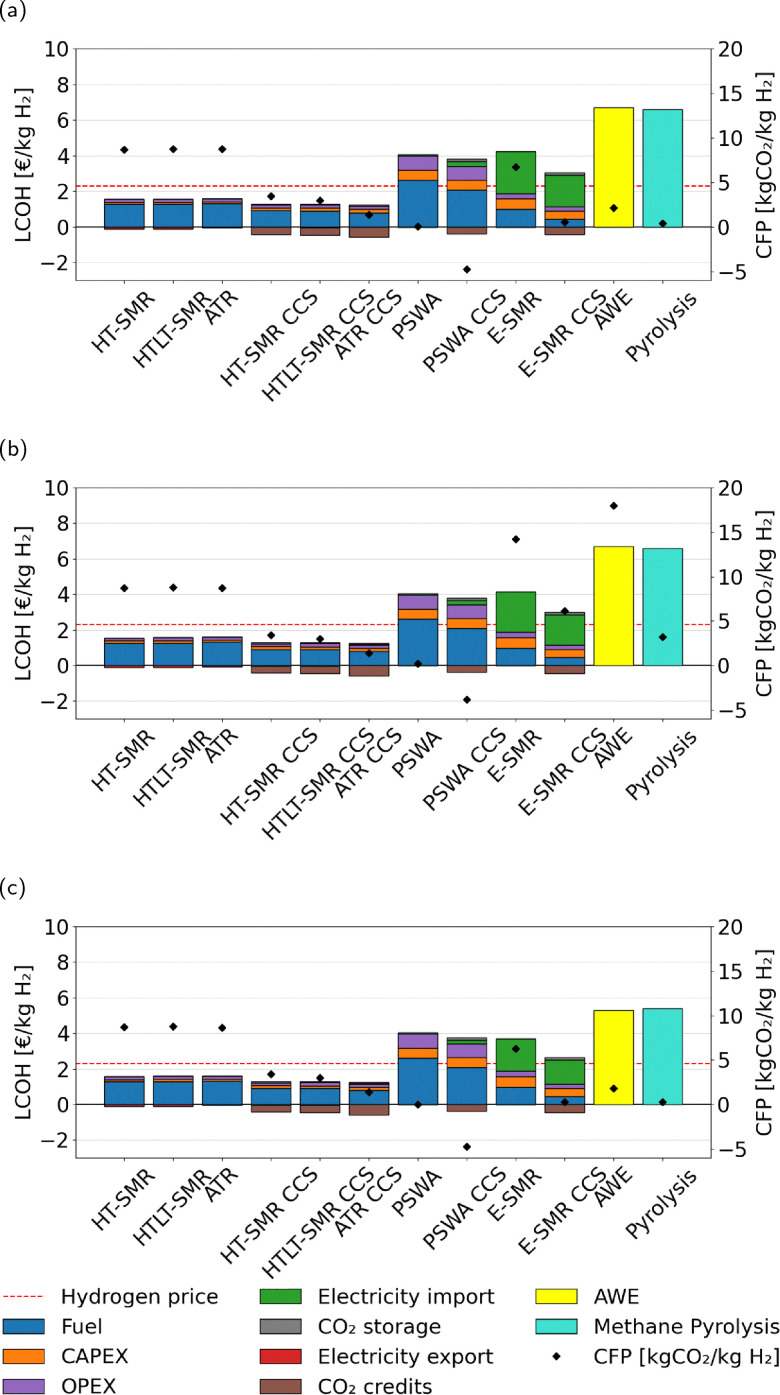
Scenario-based LCOH assessment with the use of (a) the Swiss grid, (b) the German grid and (c) the French grid for the electric supply to the processes. The red dotted line defines the threshold hydrogen market value averaged from the reference database.^87^

Fuel supply plays a key role in the assessment of hydrogen cost and footprint (Fig. 14). This effect is even more pronounced when biogas is used as feedstock. In fact, although biogas offers lower environmental impacts, the specific cost per unit of methane is higher than that of natural gas or synthetic methane (Table 3). The introduction of carbon credits significantly reduces H_2_ costs for SMR and ATR technologies. Despite higher CAPEX and OPEX, blue hydrogen (≃1.3 € per kg_H_2__) becomes cheaper than gray hydrogen (≃1.55 € per kg_H_2__) due to these incentives. The addition of LT-WGS results in slightly higher LCOH, which is due to the reduced productivity, as discussed. Nation-based scenarios show only minor variations in the cost of hydrogen produced by SMR and ATR, indicating a weak dependency on the price of electricity. However, the sale of the electricity produced in the process decreases the cost of gray hydrogen by 0.12–0.05 € per kg_H_2__, depending on the plant layout and country. As predicted using eqn (25), both biogas and e-SMR processes are highly sensitive to CAPEX and OPEX due to unfavorable scaling effects. Specifically, the biogas route is affected by the high water flow required for PSWA cleaning, while the e-SMR is affected by the water demand for syngas cooling. Unlike SMR, ATR, and biogas processes, e-SMR is the only technology whose LCOH benefits from the implementation of carbon capture. This arises from the higher volumetric flowrate due to the presence of CO_2_, which increases compression and electrical heating costs, as well as capital costs related to the recycle stream compressor. This growth in LCOH is not leveled by membrane deployment in the CCS e-SMR configuration. Both biogas (approximately 3.8–4.0 € per kg_H_2__) and e-SMR (2.65–4.0 € per kg_H_2__) are highlighted as economically more attractive than alkaline water electrolysis and methane pyrolysis for all countries. As expected, e-SMR shows the strongest dependence on the price of electricity, with the latter accounting for more than half of the required expenses. Among the three national scenarios, similar LCOH values are obtained for Swiss and German grids due to comparable average electricity costs. Better results are observed for the French grid, where the lower electricity cost reduces e-SMR with CCS to about 2.65 € per kg_H_2__, making it the most competitive among the innovative technologies. The French grid thus offers the best techno-economic and environmental performance, whilst Germany shows the worst results in both aspects, and Switzerland combines low carbon intensity with very high electricity costs. In addition to the higher convenience from an economical point of view, e-SMR with membrane-based carbon capture appears to be the most suitable technology for the electrified production of H_2_, when electricity with high footprint is supplied, as in the case of Germany. This results in a carbon footprint of H_2_, which is roughly equal to one third of that related to alkaline water electrolysis in the same country. Finally, a negligible impact is caused by the cost related to CO_2_ storage.

### 3.3 Dynamic LCOH assessment for e-SMR technology

**Fig. 15:**
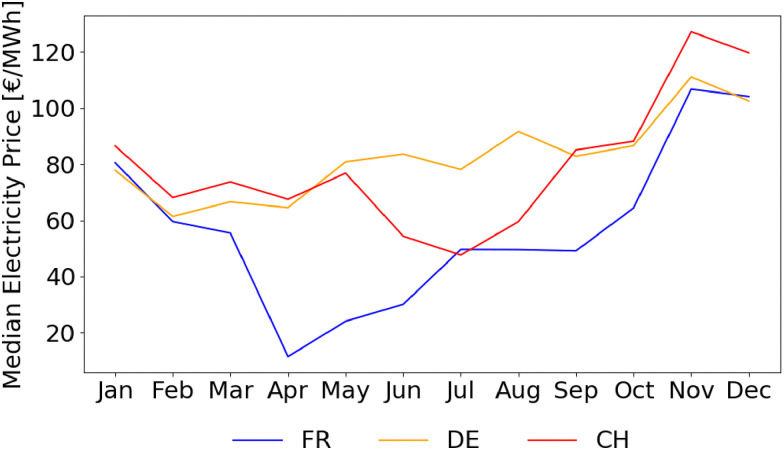
Median monthly electricity prices in 2024 in France (FR), Germany (DE) and Switzerland (CH).

**Fig. 16:**
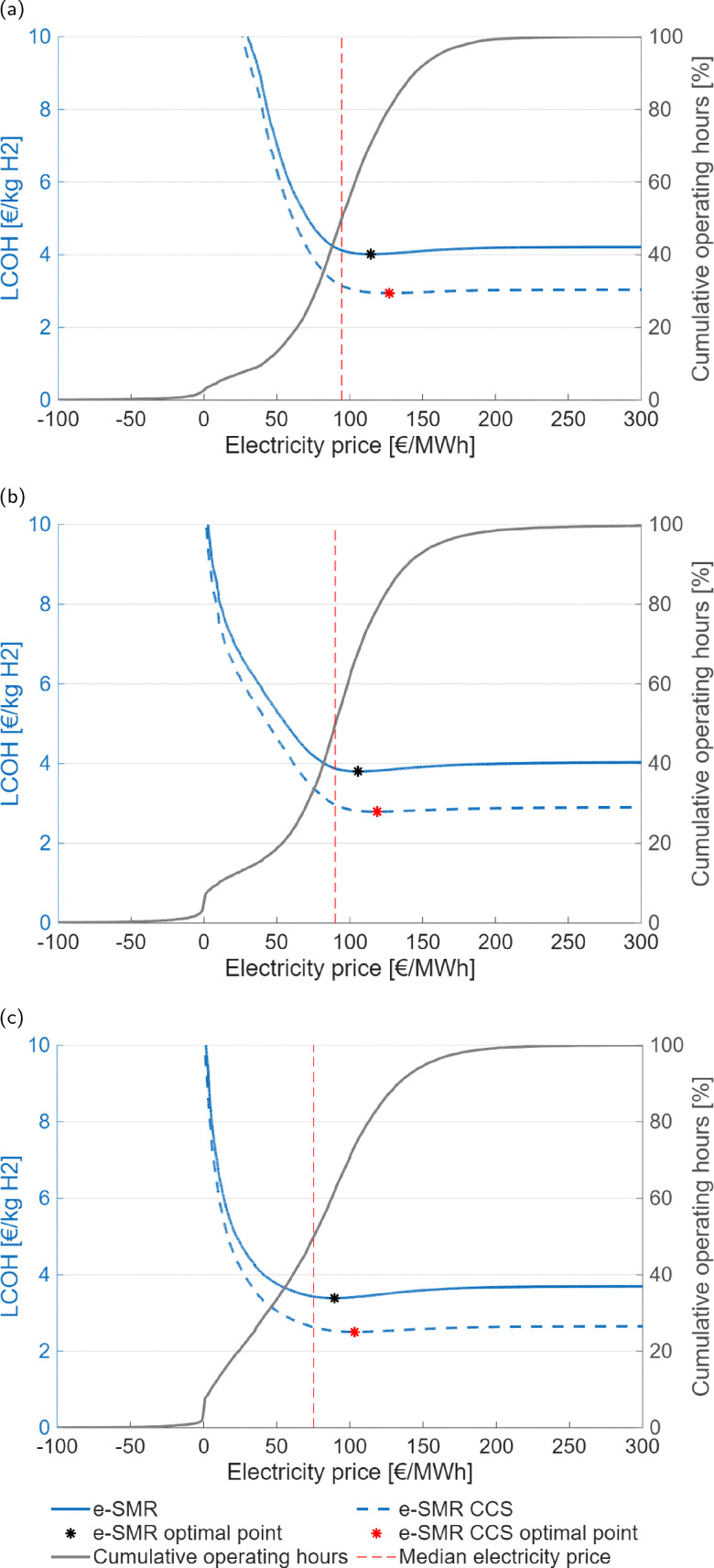
Scenario-based dynamic LCOH assessment with the use of (a) the Swiss grid, (b) the German grid and (c) the French grid for the electric supply to the processes.

The reduced start-up and shutdown times of the e-SMR technology make it a promising candidate for intermittent operation, allowing the exploitation of periods with more favorable electricity prices. As discussed in previous sections, this behavior is directly related to the high temporal and seasonal variability of the electricity (Fig. 15, A.13–A.15), driven by fluctuations in both supply and demand. Under the given considerations, electricity prices for the period of January 2023–October 2025 are analyzed and sorted to evaluate the resulting trends in hydrogen production cost according to the hours of operation. CAPEX and fixed OPEX (as catalyst cost and labor costs) are scaled to account for the 90% capacity factor adopted in previous assessments. Such an operational strategy requires reliable day-ahead electricity price forecasts to properly schedule plant operation, as well as rapid start-up procedures. In this regard, From *et al.*^27^ reported a start-up time of approximately 2.6 h to reach steady-state conditions, significantly lower than the start-up time required for the conventional SMR process. Fig. 16 illustrates consistent trends among the different scenarios. LCOH is initially high and gradually decreases as the electricity price approaches its median value, reaching a minimum, corresponding to the optimal LCOH, after approximately 60–69% and 73–78% of the available hours for e-SMR and e-SMR with CCS, respectively. This behavior can be attributed to the lower sensitivity of e-SMR with the CCS configuration to the price of electricity (Fig. 14), resulting from its reduced electrical demand. Beyond the minimum point, the LCOH increases again due to the unfavorable electricity cost, indicating that plant shutdown becomes economically preferable to continued operation. The optimal LCOH values follow the same trend observed in the steady-state assessment: France exhibits the most favorable performance, with 3.39 and 2.50 € per kg_H_2__, while the higher electricity prices of the Swiss grid result in the least competitive results, with 4.01 and 2.94 € per kg_H_2__ for the base and CCS cases, respectively. These results could be further optimized by scheduling maintenance activities during planned shutdown hours, rather than specifically allocating additional downtime for maintenance purposes. Different trends are observed in the distribution of cumulative operating hours among the different countries. Due to the substantial and continuously operating nuclear baseload, the French electricity cost exhibits only a minor dependence on hourly fluctuations associated with renewable energy surplus. This behavior results in an almost linear increase once positive prices are reached (Fig. 16), accompanied by a negligible number of hours with negative prices. In contrast, the larger share of renewables in the German grid leads to a higher frequency of negative-priced hours, which are particularly favorable for e-SMR operation. Moreover, the cumulative distribution of operating hours follows a markedly different, sigmoidal trend rather linear. This behavior yields the most significant improvement in terms of LCOH reduction compared to static assessment, reaching approximately 0.36 and 0.20 € per kg_H_2__ for the base and CCS cases, respectively. Despite a considerable fraction of economically non-viable hours, the most favorable LCOH values remain lower than those observed in other scenarios, highlighting the suitability of e-SMR operation under these conditions. Indeed, since the most favorable hours are concentrated in a narrow time interval, a very quick startup time is beneficial to their exploitation. As in the static assessment, the Swiss grid exhibits an intermediate behavior, resulting from both its comparatively higher electricity price and its moderate dependence on renewable generation. Thus, intermittent operations would be highly beneficial for this type of process, leading to remarkably low LCOH values, with reductions of approximately 0.1–0.3 € per kg_H_2__ compared to continuous operation discussed in previous sections. However, more attention should be devoted to improving the accuracy of electricity price forecasting and minimizing plant startup times, which remain critical factors for the practical implementation of such flexible operation strategies.

## Conclusions

The urgent need to develop low-carbon productive routes to replace the use of conventional processes for hydrogen production calls for the development of technologies that use biogenic feedstock or electricity. In this work, conventional SMR and ATR processes were compared with novel technologies, such as biogas reforming and e-SMR in terms of process efficiency, product carbon footprint, and levelized cost of hydrogen (LCOH), assessed following modeling and simulation of all the processes. The limited size of biogas and renewable electricity production plants implies a low-scale and distributed implementation of the biogas reforming and electrified SMR. This results in lower process efficiencies, primarily due to the limited potential for heat and process integration, different from large-scale SMR and ATR systems where process optimization leads to efficiencies between 76 and 81%. The addition of CCS partially mitigates this drawback: the higher LHV of the tail gas and the reduced volumetric flow rate to the reactor lead to improvement in the process performance. This allows increasing the efficiency of biogas reforming from 56–68% to 65–69% and the effectiveness of e-SMR from 59% to 71%. SMR and ATR emit roughly 8.6–8.7 kg_CO_2__ kg_H_2__^−1^ and 1.2–3.4 kg_CO_2__ kg_H_2__^−1^, without and with carbon capture, respectively. Small-scale technologies demonstrated to lead to substantial improvements from an environmental point of view because they allow the valorisation of renewable feedstock. The biogenic origin of biogas reduces the carbon emissions, which are only due to the use of grid-derived electricity. Furthermore, negative emissions are observed where CCS is implemented, with the permanent capture of biogenic carbon dioxide. In contrast, the carbon balance of the e-SMR strongly depends on the electricity source. Hence, the carbon footprint of e-SMR was analyzed using different country-based grid scenarios. The process shows great benefits from the use of low-carbon electric energy, as in the case of the Swiss grid, while CFP exceeds the benchmark values of HT-SMR in case wider fossil shares are present in the energy mix, as for Italian, German, and Chinese grids. Similar behavior is observed in case CCS is deployed, as only under the Swiss scenario, the carbon emissions are successfully reduced compared to HT-SMR with carbon capture. As a result, e-SMR shows promising results for emission abatement only if the process employs low-carbon electricity. From an economic point of view, the benchmark SMR and ATR processes have been demonstrated to be the most viable, resulting in LCOH of approximately 1.6 € per kg_H_2__ and 1.3 € per kg_H_2__ for the base layout and implementation of CCS, respectively. Despite the higher capital and operating costs due to the carbon capture unit and CO_2_ compression, the introduction of carbon credits for permanently stored CO_2_ results in lower product costs for ATR and SMR with CCS, compared to standard processes. Biogas and e-SMR LCOH is affected by the small scale of the processes, which results in a higher impact of CAPEX and OPEX on the overall costs because of the scale law. In addition, biogas is influenced by the higher production price of the feed compared to natural gas, leading to LCOH of 3.8–4.0 € per kg_H_2__, with a slight influence of both price of electricity and the addition of CCS. Real case scenarios are analyzed in terms of electricity prices for the different countries, to understand how the latter impacts e-SMR LCOH. French, German and Swiss day-ahead prices from EPEXSPOT were averaged for the time series from Jan 2023 to Oct 2025. German and Swiss scenarios showed similar results in terms of LCOH, with values of about 4.2 and 3.0 € per kg_H_2__ for the base case and for the CCS case, respectively. The most competitive hydrogen costs in the small scale are demonstrated for the French case, as the lower electricity price leads to 3.7 and 2.6 € per kg_H_2__ with and without the addition of CCS, respectively. For the electrified reforming case study, CCS is shown to be beneficial to the hydrogen price regardless of carbon credits, different from other processes. Both biogas and e-SMR are assessed as more economically convenient compared to alkaline water electrolysis and methane pyrolysis, regardless of the country. An electricity mix like France is hence a suitable condition for the development of e-SMR technology, due to both low electricity price and low-carbon footprint, while the case of Germany is the worst under an economical and environmental scenario. A dynamic analysis of real electricity costs was performed for e-SMR, in order to understand the benefits and feasibility of intermittent operation for this kind of technology, aimed at taking advantage of the most economically convenient hours. The shutdown of the plant when the price is the highest was found to be beneficial to the economic performance of the process, resulting in a reduction of 0.2–0.4 € per kg_H_2__ and 0.1–0.2 € per kg_H_2__ for the base and CCS layouts, respectively. This effect is more pronounced when CCS is not present, because the higher dependence of the base layout was shown to be the best performing, with 3.4 and 2.5 € per kg_H_2__ for the base and CCS case, respectively. Germany showcases the most significant benefits from this dynamic operation, due to the high fraction of negative and low priced hours related to renewable energy excess, as demonstrated by the sigmoidal trend of the cumulative operating hours. Finally, the Swiss scenario is influenced by the nuclear and hydroelectric baseload, resulting in lower price fluctuations and in overall higher prices. Despite future challenges consisting of the scale-up of biogas reforming and electrified steam methane reforming, both technologies are beneficial to the mitigation of carbon emissions. Moreover, promising levelized cost of hydrogen is assessed for the latter, with the best performances observed in the case the e-SMR with CCS is fed by the French electricity grid. Additionally, further economical improvements are foreseen in case e-SMR is operated intermittently. For these reasons, the two routes are demonstrated as considerable candidates for the future decarbonization of hydrogen production. This study demonstrates that e-SMR and biogas reforming can be promising alternatives to electrolysis for sustainable H_2_, as they require a lower amount of electricity and can better adapt to oscillations in the electricity price. This can be a key for the implementation of new, less carbon intensive technologies in the near future. However, the sustainability of these technologies depends strongly on the application of consistent carbon management policies, which should allow permanent storage of CO_2_ and strive for the decrease in the carbon footprint of the electricity grid.

## Author contributions

Giulio Martinoli: conceptualization, methodology, formal analysis, investigation, data curation, methodology, visualization, writing – original draft, and writing – review and editing. Emanuele Moioli: conceptualization, methodology, formal analysis, investigation, writing – original draft, writing – review and editing, supervision, and funding acquisition.

## Conflicts of interest

There are no conflicts to declare.

## Nomenclature

### Abbreviations

AWE: Alkaline water electrolysis
ASU: Air separation unit
ATR: Auto-thermal reforming
BEC: Bare erected cost
BEC_cpr_: Compressor bare erected cost
BEC_vessels_: Bare erected cost for vessels
BEC_HE_: Bare erected cost for heat exchangers
CAPEX: Annual capital expenditures
C: Contingencies
CCS: Carbon capture and storage
CEPCI: Chemical engineering plant cost index
CEPCI_2024_: Chemical engineering plant cost index in June 2024
CEPCI_y_: Chemical engineering plant cost index updated to year y
CFP: Carbon footprint
CH: Switzerland
CHI: China
CHP: Combined heat power
DE: Germany
EPC: Engineering, procurement and construction costs
e-SMR: Electrified steam methane reforming
FR: France
GWP: Global warming potential
HDS: Hydrodesulphurization
HT-WGS: High temperature water–gas shift
IC: Plant indirect costs
I: Insurance cost
IT: Italy
L: Catalyst lifetime
LCOH: Levelized cost of hydrogen
LCOH_CH_: Levelized cost of hydrogen in Switzerland
LCOH_FR_: Levelized cost of hydrogen in France
LCOH_DE_: Levelized cost of hydrogen in Germany
LHV: Molar lower heating value
LP: Low-pressure
LT-WGS: Low temperature water–gas shift
M: Maintenance cost
MDEA: Methyldiethanolamine
NC: Number of components in the natural gas mixture
NR: Number of reactors
O/C: Oxygen-to-carbon ratio
OPEX: Annual operating expenditures
PSA: Pressure swing adsorption
PSWA: Pressure swing water absorption
S/C: Steam-to-carbon ratio
SMR: Steam methane reforming
TDPC: Total direct plant cost
TIC: Total installation cost
TOC: Total overnight cost
TRL: Technology readiness level
VPSA: Vacuum pressure swing adsorption
WGS: Water–gas shift

### Symbols

*α*
: Splitting factor
*β*
: Purge ratio
Δ*H*^0^_r_: Standard reaction enthalpy
*χ*_CO_
: Carbon monoxide conversion
*η*
: Energy plant efficiency
*η*_CO_2__
: Carbon dioxide capture efficiency
*η*_el_
: e-Reformer energy efficiency
*η*_LHV_
: LHV-based plant efficiency
*η*_PSA_
: Hydrogen recovery efficiency in the PSA unit
*η*_VPSA_
: Hydrogen recovery efficiency in the VPSA unit
*C*_2024_
: Equipment cost updated to Jun 2024
*C*_0,CO_2__
: Reference annual CO_2_ storage cost
*C*_0,labour_
: Reference annual labour cost
*C*_cat_
: Annual catalyst cost
*C*_cw_
: Cooling water volumetric cost
*C*_el_
: Annual electricity cost
*C*_el,avg_
: Average nation-based electricity cost
*C*_el,CH_
: Annual electricity cost in Switzerland
*C*_el,DE_
: Annual electricity cost in Germany
*C*_el,FR_
: Annual electricity cost in France
*C*_el,*n*_
: Electricity price for *n*-th hour of the time series
*C*_fixed_
: Fixed plant costs for the dynamic operation
*C*_feed,LHV_
: LHV-specific feedstock cost
*C*_fuel_
: Annual feedstock cost
*C*_HDS_
: Volumetric HDS catalyst cost
*C*_*i*_
: *i*-th equipment cost
*C*_labour_
: Annual labour cost
c/m: CO_2_ to MDEA molar ratio
*C*_ref_
: Volumetric steam reforming catalyst cost
*C*_WGS_
: Volumetric WGS catalyst cost
*C*_water_
: Annual water cost
*C*_y_
: Equipment cost updated to year y
CO_2credits_: Mass-specific carbon credits
CO_2profit_: Annual incentives from carbon credits
CFP_AWE_: Alkaline water electrolysis carbon footprint
CFP_grid_: Electric grid carbon footprint
CFP_pyrolysis_: Methane pyrolysis carbon footprint
*F*_0,feed_
: Reference plant feedstock
*F*_CO_2__
: Captured carbon dioxide molar flux
*F*_feed_
: Feedstock molar flux to the plant
*F*_H_2__
: Produced hydrogen molar flux
*F*_NG_
: Natural gas molar flux to the plant
*F*_NG,C_2*n*_H_2*n*+2__
: Hydrocarbon molar flux in natural gas stream
*F*_NG,fuel_
: Natural gas molar flux to the reformer furnace side
*F*_NG,ref_
: Natural gas molar flux to the reformer tube side
*F*_O_2__
: Oxygen molar flux
*F*_purge_
: Purge molar flux
*F*_tail_
: Tail gas molar flux
*F*_w_
: Steam molar flux
*f*
: Scaling factor
*h*_cost,*i*_
: Cumulated operational plant costs for the *i*-th hour
*Ḣ*_out,ref_
: Reformer product enthalpy flux
*Ḣ*_in,ref_
: Reformer feed enthalpy flux
*h*_prod,*i*_
: Cumulated hydrogen production for the *i*-th hour
*h*_rev,*i*_
: Cumulated plant revenues for the *i*-th hour
*i*
: Position of the *i*-th hour in the time series
*L*
: Catalyst lifetime
LCOH_AWE_: LCOH for AWE technology
LCOH_AWE,40_: AWE LCOH with electricity price at 40 € per MWh
LCOH_AWE,60_: AWE LCOH with electricity price at 60 € per MWh
LCOH_dyn,*i*_: Dynamic LCOH for the *i*-th hour of the time series
LHV_NG_: Natural gas molar-specific lower heating value
LHV_H_2__: Hydrogen molar-specific lower heating value
LHV_feed_: Feedstock molar-specific lower heating value
*m*
: Slope used for AWE LCOH assessment
*m*_0,CO_2__
: Reference mass flux of captured carbon dioxide
MW_CO_2__: Carbon dioxide molar weight
MW_H_2__: Hydrogen molar weight
*N*_hours_
: Number of plant operating hours
*N*_years_
: Plant lifetime
*n*_serie_
: Number of hours in the Jan 2023–Oct 2025 time series
*P*
: Pressure
*q*
: Intercept used for AWE LCOH assessment
*Q*_biogas_
: Heat requirements for biogas preheating
*Q*_reformer_
: Heat requirements for biogas reformer
*Q*_steam_
: Heat requirements for steam overheating
*S*_*i*_
: *i*-th equipment capacity
*S*_0_
: Reference equipment capacity
*T*_HT-WGS_
: HT-WGS unit inlet temperature
*T*_HDS_
: HDS unit inlet temperature
*T*_LT-WGS_
: LT-WGS unit inlet temperature
*T*_PSA_
: PSA unit inlet temperature
*T*_Pre-ref_
: Pre-reforming unit inlet temperature
*T*_fuel_
: Fuel temperature
*T*_flue,in_
: Flue gas temperature at the heat recovery section inlet
*T*_flue,out_
: Flue gas temperature at the heat recovery section outlet
*T*_in_
: Reformer inlet temperature
*T*_out_
: Reformer outlet temperature
*V*_HDS_
: Feed-specific HDS volume
*V*_WGS_
: Feed-specific WGS volume
*V*_ref_
: Feed-specific reformer volume
*V̇*_cw_
: Cooling water volumetric flux
*V̇*_H_2__
: Normal volumetric hydrogen flux
*V̇*_w,process_
: Process water volumetric flux
*W*
: Compressor electrical requirement
*W*_AWE_
: Mass-specific energy consumption in AWE
*w*_M_
: MDEA weight fraction CO_2_-free solution
*W*_pyrolysis_
: Mass-specific energy consumption in methane pyrolysis
*W*_H_2__
: Volumetric energy requirement for H_2_ electrified production
*Ẇ*_process_
: Electrical power produced by the process
*Ẇ*_ref_
: Electrical power required by the electrified reformer
*x*_*i*_
: *i*-th species molar fraction

## Supplementary Material

YA-005-D5YA00346F-s001

## Data Availability

The data supporting this article have been included as part of the supplementary information (SI). Supplementary information: data used for the calculations, details of the balancing procedures, results of the calculations and details on the additional configurations considered. See DOI: https://doi.org/10.1039/d5ya00346f.
