# Supplementary material for: Dynamic eco-techno-economic analysis of low-carbon hydrogen production from methane
Source: Energy Adv. 2026 Feb 2;5(3):290–314. doi: 10.1039/d5ya00346f (PMC12930395; doi:10.1039/d5ya00346f)
Supplement: YA-005-D5YA00346F-s001 [file YA-005-D5YA00346F-s001.pdf]

Supplementary Information (SI) for Energy Advances.
This journal is © The Royal Society of Chemistry 2026

A Supplementary information to 'Dynamic eco-techno-economic analysis of the sustainable hydrogen production from methane'

Validation of the methodology used

Figure A.1 shows the comparison between the results of this work and the reference values by Antonini et al[36]. Figure A.2 compares the LCOH calculated in this work with literature data[29].

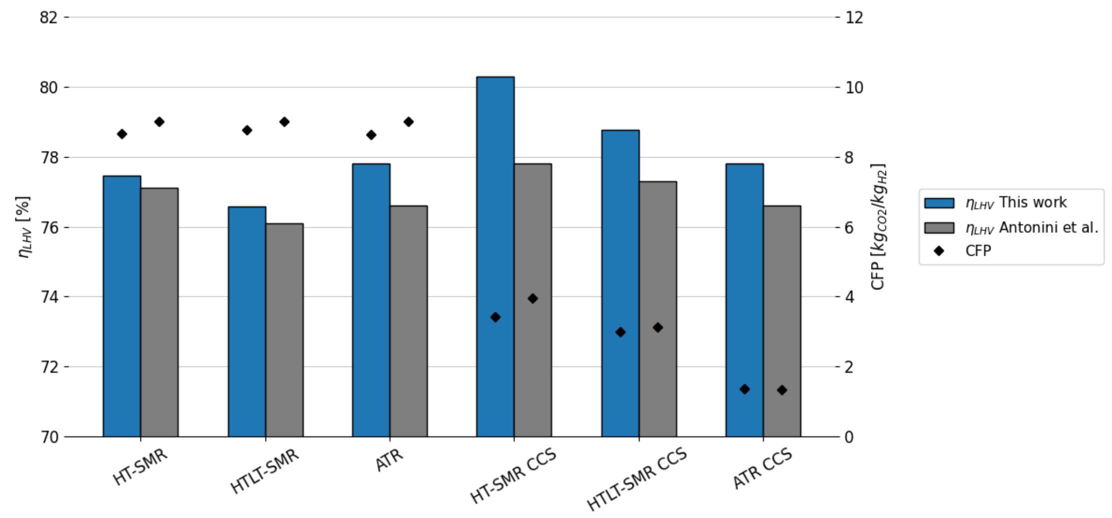

Fig. A.1 Validation of the plant simulations performed in this work against with a reference paper concerning SMR and ATR[36], in terms of LHV-based efficiency and carbon footprint for SMR and ATR processes.

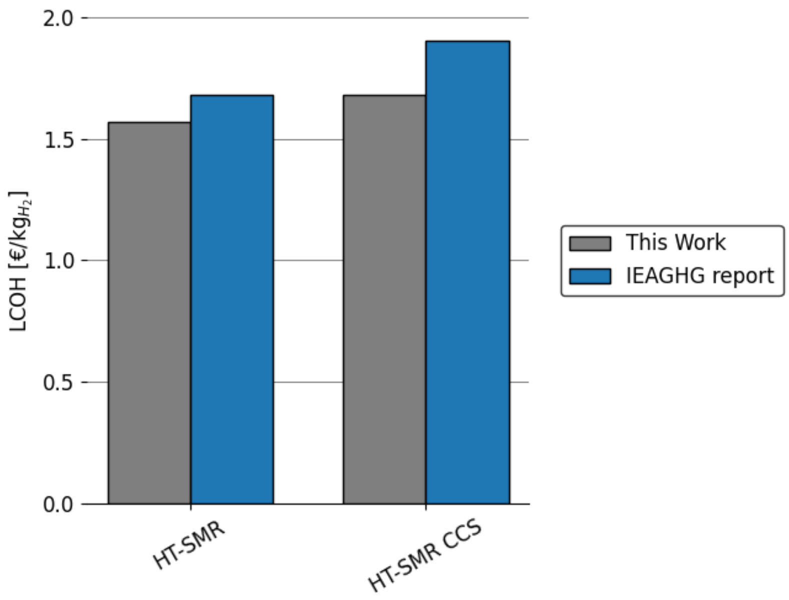

Fig. A.2 Validation of LCOH assessment without carbon credits for CCS for grey and blue hydrogen production with HT-SMR, through a comparison with reference work[29].

Additional details of the process simulations

This section reports the details concerning the process simulations, to support the reproducibility of the results of this study.

Figure A.3 shows the variation of CO conversion and outlet composition for different inlet temperatures.

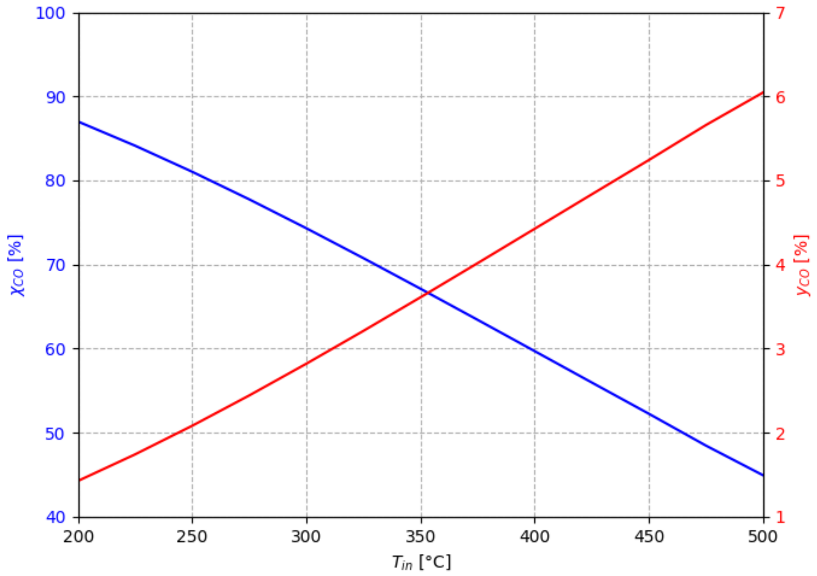

Fig. A.3 Water-gas shift equilibrium conversion (left y-axis) and outlet CO molar fraction (right y-axis) for the feed reported in Table 2, at different reactor inlet temperatures.

Figures A.4-A.5 report the sensitivity analysis underlying the selection of pressure, temperature and S/C ratio, aimed at optimizing LHV-based efficiency and split factor between the gas stream directed to the reformer and the fraction used to produce heat for the reformer itself, for the steam reforming reference plant. Additionally, figure A.6 shows the effect of the inlet temperature of the fuel on the efficiency and on the split factor.

Figure A.7 reports the sensitivity analysis of the influence of O/C, S/C ratios and pressure on the resulting process efficiency for the ATR plant. The assessment of the effect of the O/C ratio is further elaborated in figure A.8, showing the optimal value to employ in the plant operation.

Figure A.9 shows the effect of purge ratio on the energy cost of the product with and without CCS. Additionally, Figure A.10 shows the effect of purge ratio on the H_2 productivity with and without CCS.

Figure A.11 reports the sensitivity analysis performed to determine the optimal operating temperature for the e-SMR plant as a function of pressure and outlet temperature. Figure A.12 shows the same sensitivity for the case with implementation of CCS. Figures A.13, A.14 and A.15 show the seasonal variation of day-ahead price of electricity for the three countries selected for the geographic sensitivity analysis in 2024.

Figure A.16 shows the trade off between plant efficiency and carbon footprint, as a function of CO conversion in the WGS section and CO_2 capture efficiency through amine process.

Figure A.17 describes the available heat remaining at the outlet, as a function of CO conversion in the WGS section and CO_2 capture efficiency through amine process.

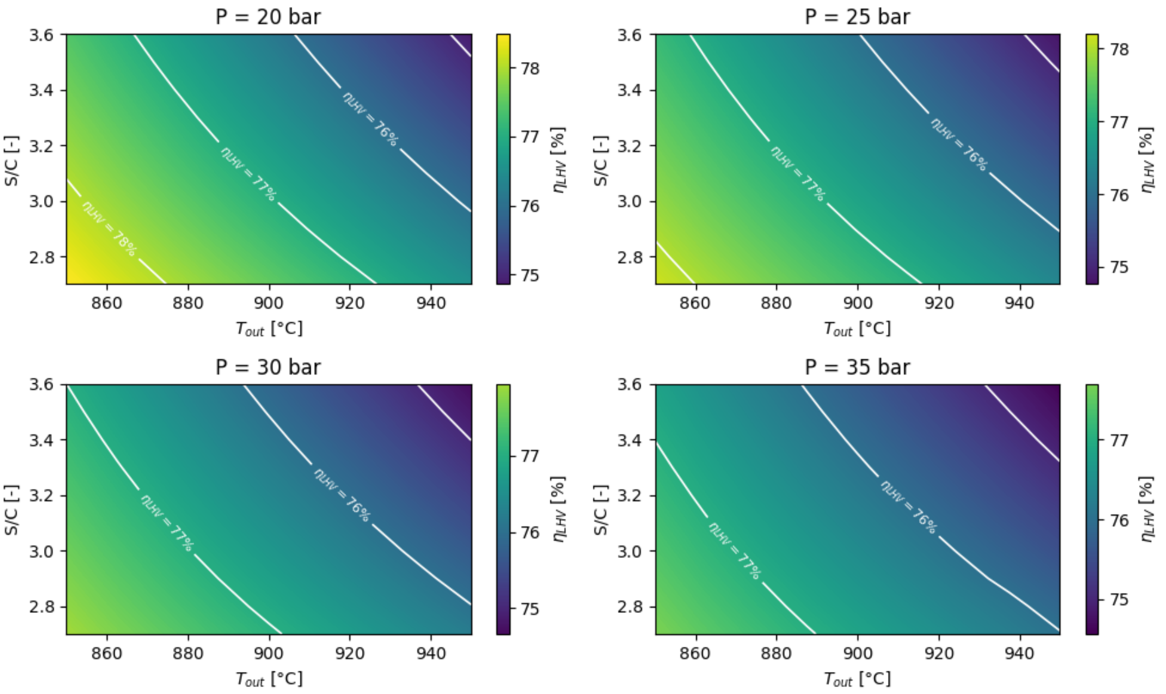

Fig. A.4 Sensitivity analysis of pressure, reformer outlet temperature and S/C ratio influence on LHV-based plant efficiency (η_{LHV}) in HT-SMR process simulation.

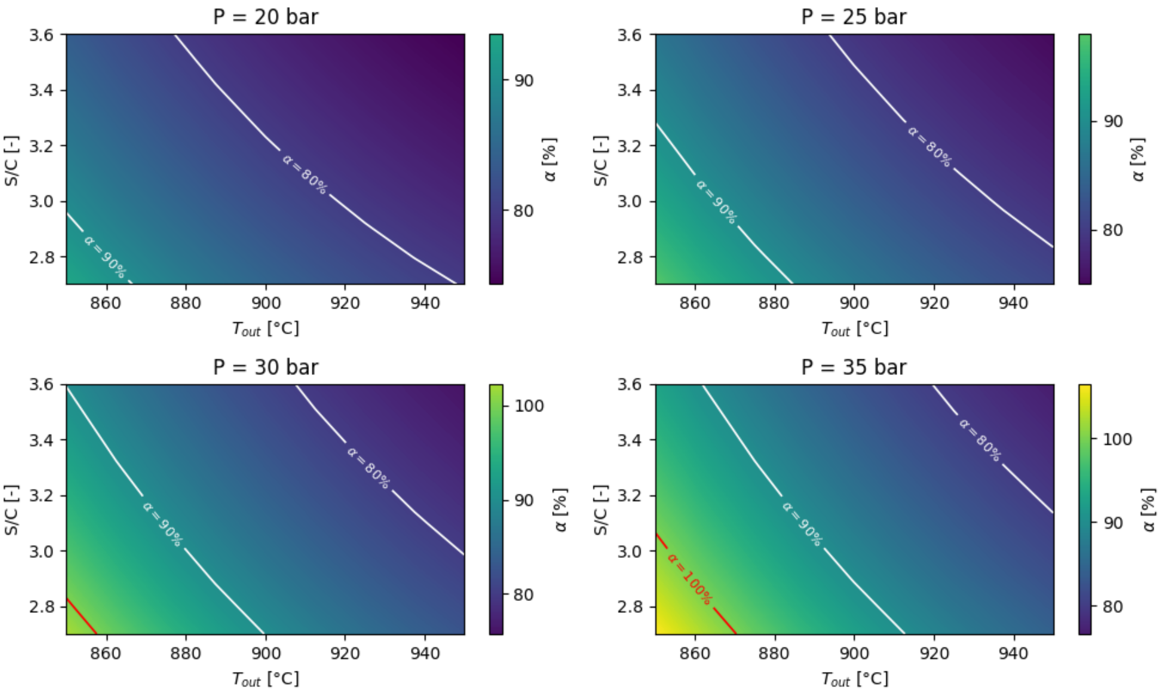

Fig. A.5 Sensitivity analysis of pressure, reformer outlet temperature and S/C ratio influence on splitting factor α in HT-SMR process simulation. The red dotted line highlights physically unfeasible values ($\alpha > 100\%$).

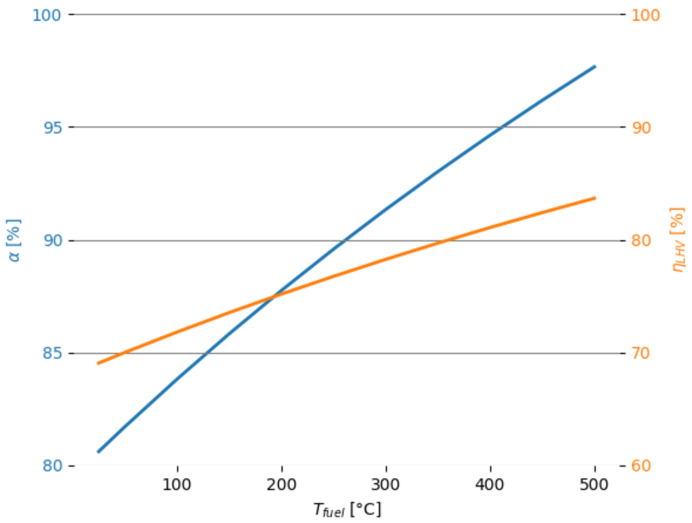

Fig. A.6 Fuel temperature (T_{fuel}) sensitivity analysis on LHV-based process efficiency (η_{LHV}) and natural gas splitting ratio (α) in HT-SMR plant simulation.

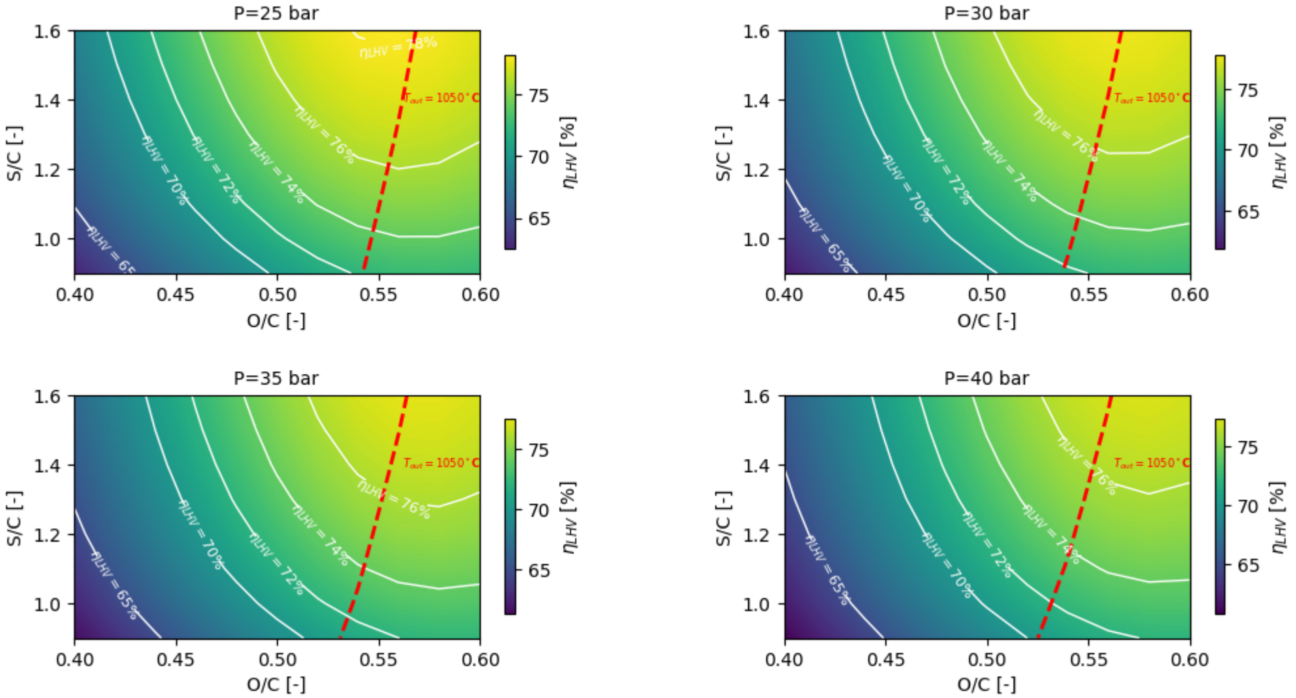

Fig. A.7 Sensitivity analysis of O/C, S/C and P influence on LHV-based process efficiency (η_{LHV}) in ATR plant simulation. The red dotted line indicates the maximum allowable outlet temperature of 1050 °C, increasing towards the right.

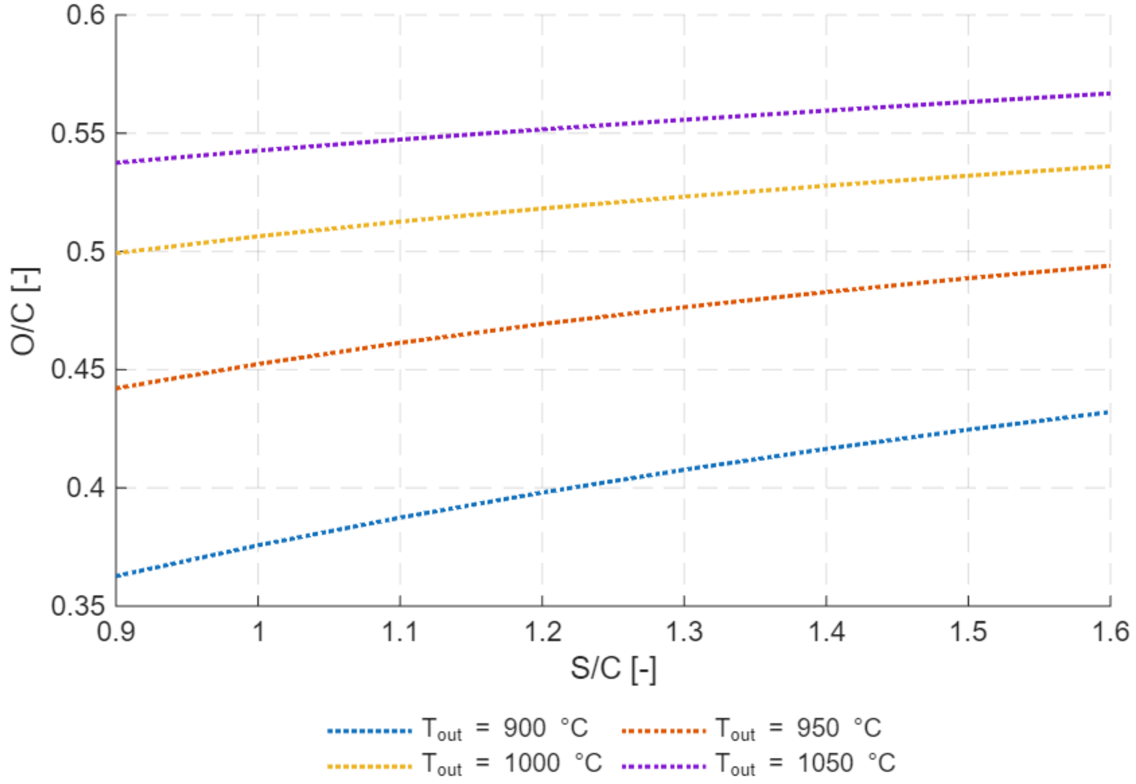

Fig. A.8 Stoichiometric O/C assessment in an ATR plant operating at 30 bar, at varying reformer outlet temperatures and S/C ratios.

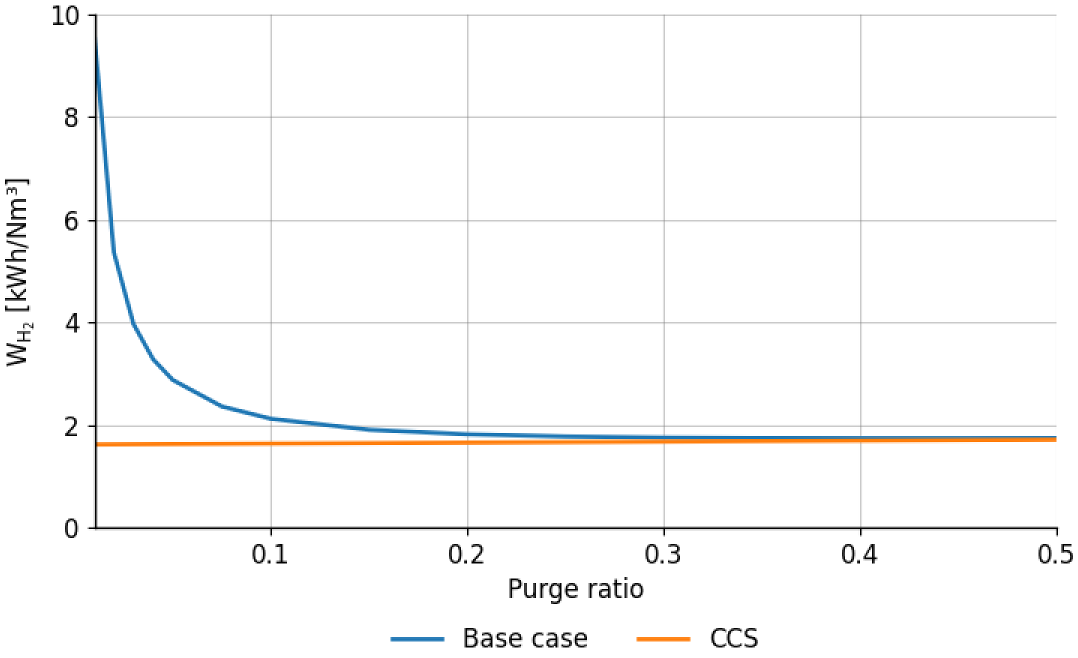

Fig. A.9 Energy cost of hydrogen as a function of the purge ratio for e-SMR process.

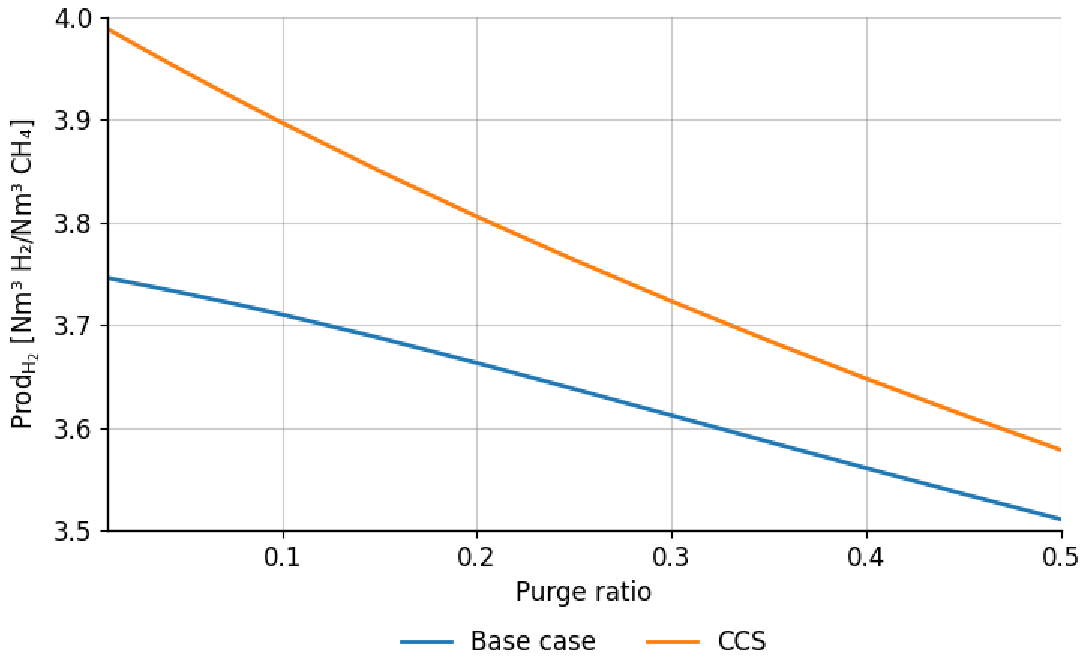

Fig. A.10 Hydrogen specific productivity as a function of purge ratio for e-SMR process.

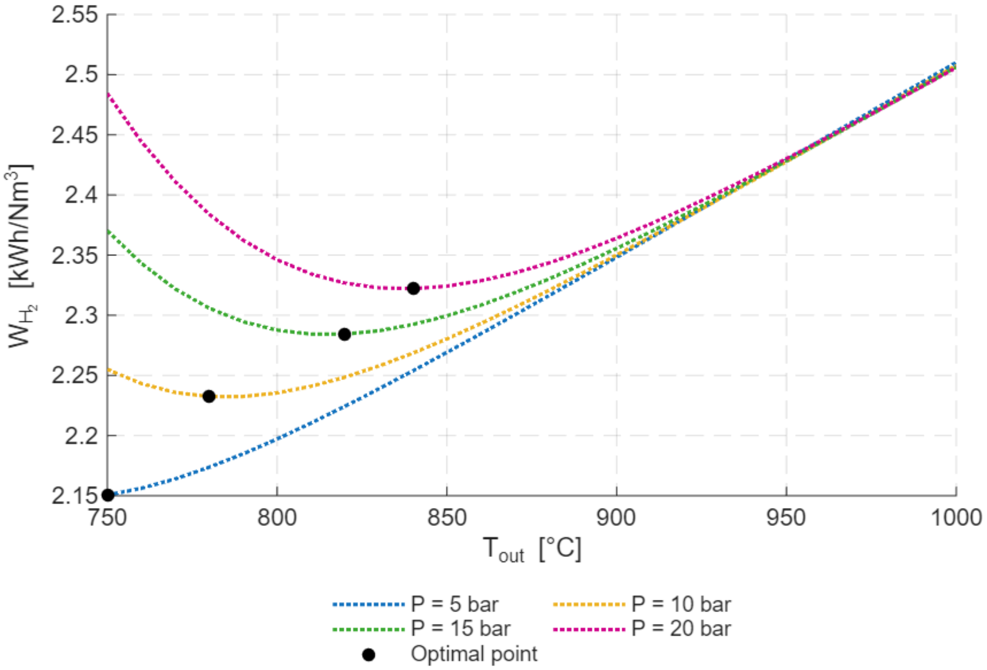

Fig. A.11 Sensitivity analysis of pressure and outlet temperature influence on hydrogen electric cost without CCS implementation. The optimal operating temperature for each pressure line is pointed out as a black dot.

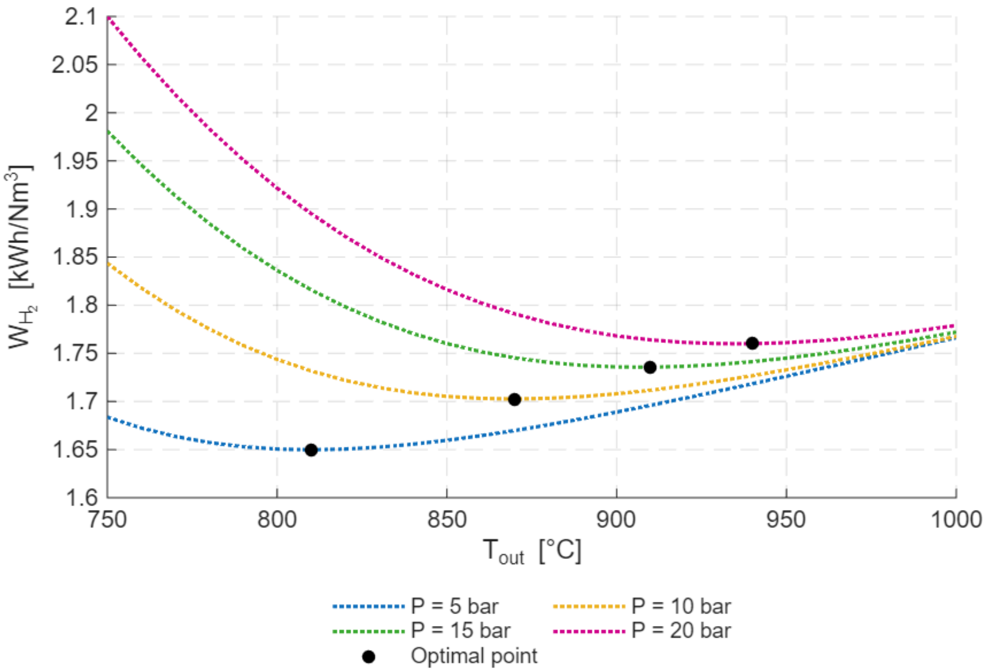

Fig. A.12 Sensitivity analysis of pressure and outlet temperature influence on hydrogen electric cost with CCS implementation. The optimal operating temperature for each pressure line is pointed out as a black dot.

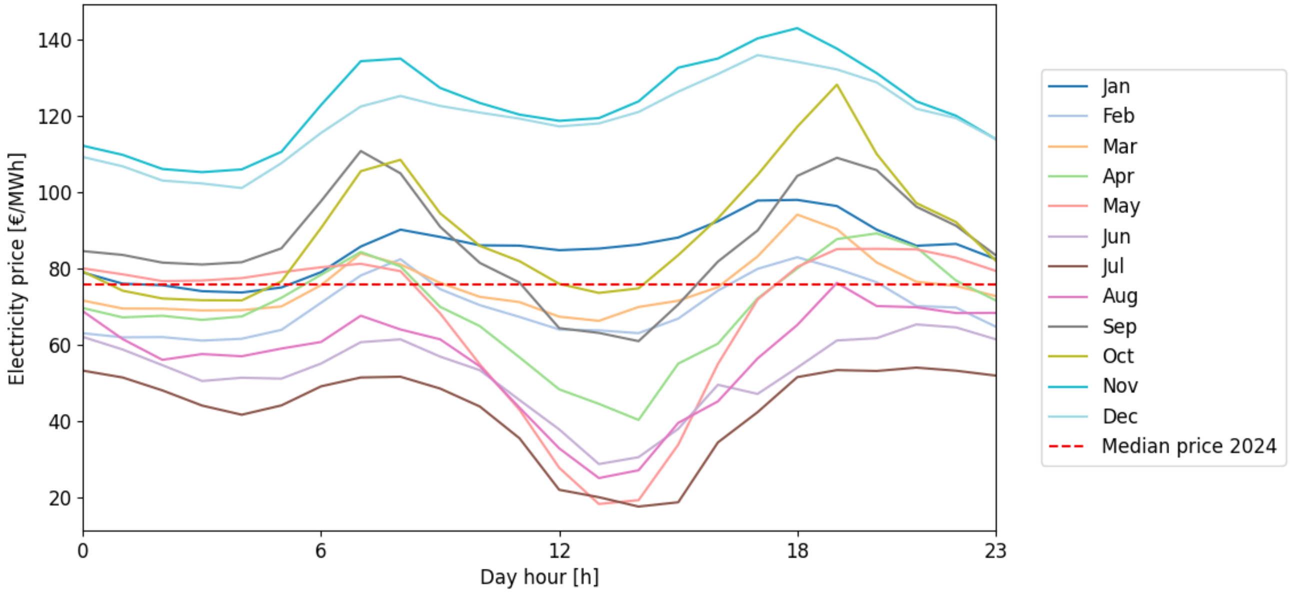

Fig. A.13 Median hourly electricity price in Switzerland in 2024, ordered by month

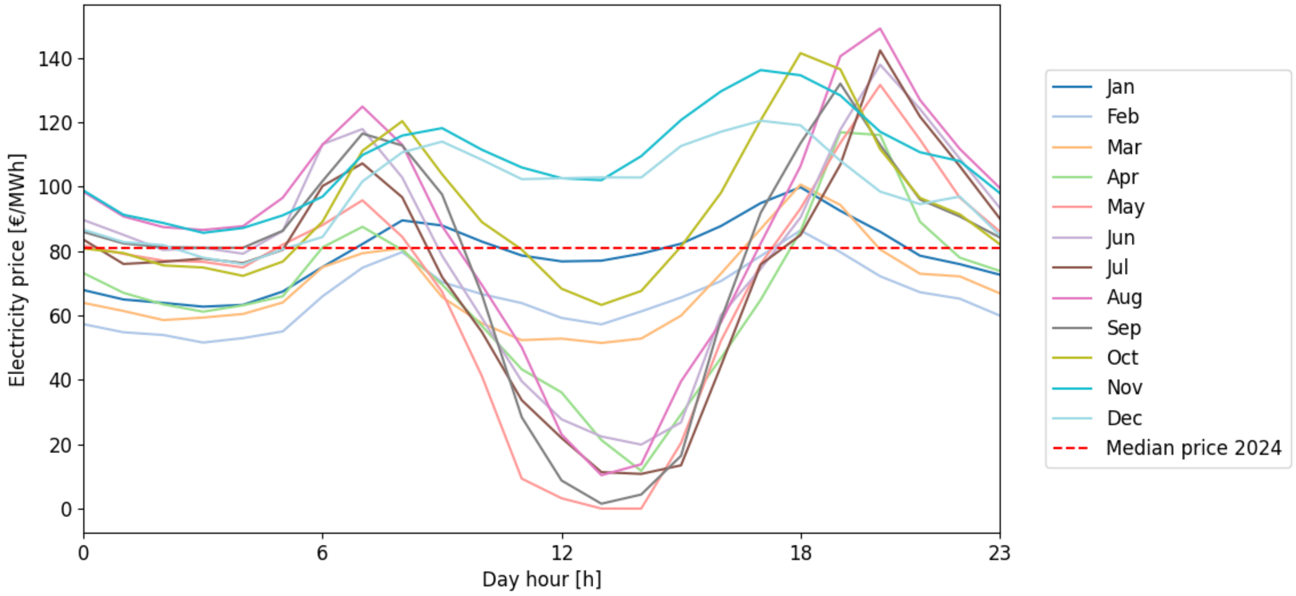

Fig. A.14 Median hourly electricity price in Germany in 2024, ordered by month

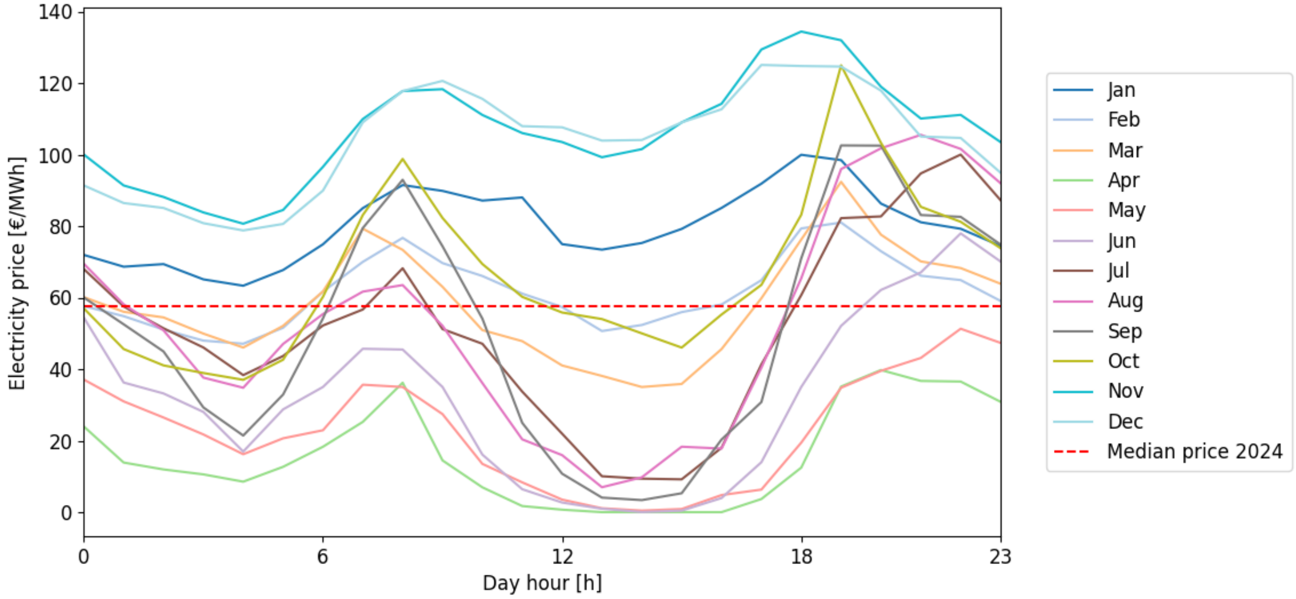

Fig. A.15 Median hourly electricity price in France in 2024, ordered by month

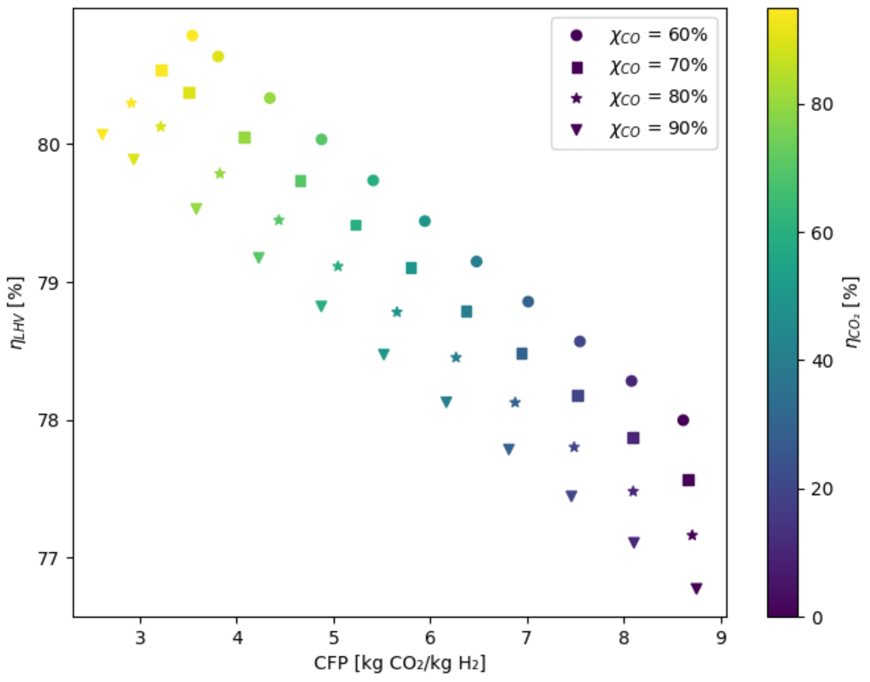

Fig. A.16 Trade-off between LHV-based plant efficiency (η_{LHV}) and hydrogen carbon footprint (CFP), with respect to CO conversion via WGS (χ_{CO}) and CO_2 unit capture efficiency (η_{CO_2}).

.

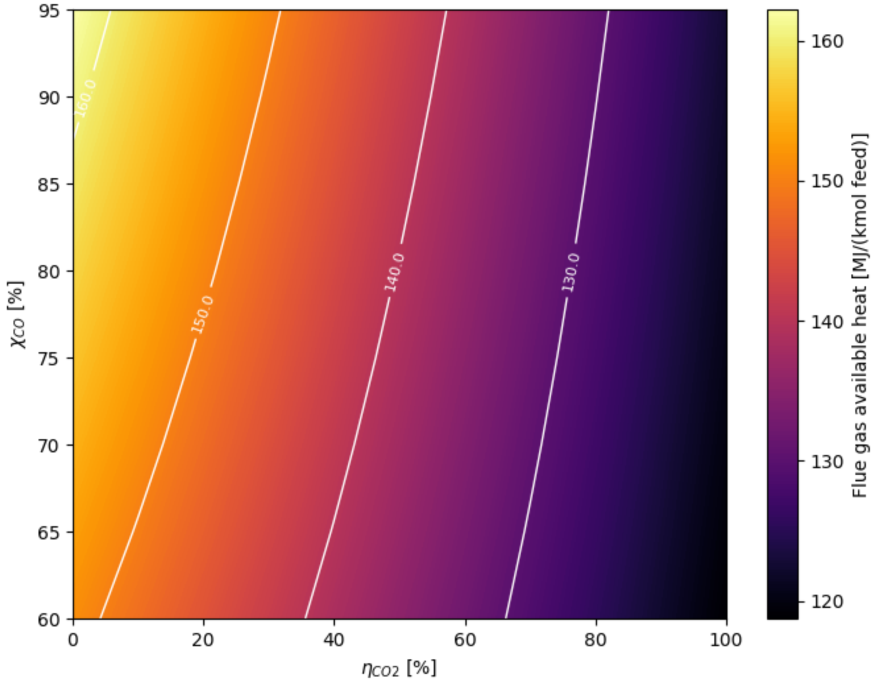

Fig. A.17 Analysis of available heat derived from flue gases cooling to 130 ˚C as a function of CO conversion via WGS catalysis (χ_{CO}) and CO_2 unit capture efficiency (η_{CO_2}).

Raw calculation data

The following tables report the main input and output data of this study.

Table A.7 summarizes the process variables selected in the simulations reported in this study.

Table A.8 summarizes the inlet and outlet fluxes and process metrics for LT-SMR, HT-SMR, ATR, FLOX®, PSWA and e-SMR configurations, using Italian grid as a source of electricity.

Table A.9 summarizes resulting metrics used for SMR and ATR processes validation with previous works.

Table A.10 reports the main results in terms of yearly costs, electricity costs and LCOH for HT-SMR, HTLT-SMR, ATR, FLOX®, PSWA and e-SMR configurations, each divided into Base and CCS cases.

Table A.11 reports in tabular form the results of the analysis of reference processes, electrolysis and methane pyrolysis.

Table A.12 shows the results of the dynamic analysis.

Table A.13 summarizes the price assumptions adopted within this work.

Table A.14 shows the median monthly electricity prices in 2024, elaborated from the raw electricity price time series.

Table A.7 Summary of process variables for SMR, ATR, FLOX®, PSWA and e-SMR configurations. Feed is the input natural gas or biogas or methane to the plant, P is the operating pressure, S/C and O/C are defined in Equations 6, 11. Subscripts in T variables indicate the unit to which the value is the inlet temperature, apart from T_{in} and T_{out} which are the reformer inlet and outlet temperatures, T_{amb} which indicates ambient temperature at the plant battery limits, T_{fuel} which refers to the tail gas and air mixture, $T_{flue,in}$ and $T_{flue,out}$ which define flue gas inlet and outlet temperature in heat recovery section.

η stands for the percentage recovery efficiency of the unit defined by the subscript. Excess air % refers to the quantity used for tail gas and fuel combustion. γ indicates the percentage of product hydrogen to be recycled to HDS section for natural gas pre-treatment.

Variable	SMR	ATR	FLOX®	PSWA	e-SMR
P [bar]	30	30	15	24	10
S/C [-]	2.7	1.6	6	3	3
O/C [-]	–	0.56	–	–	–
T_{amb} [°C]	20	20	20	20	20
T_{HDS} [°C]	400	400	–	–	–
$T_{Pre-ref}$ [°C]	500	500	–	–	–
T_{in} [°C]	600	700	700	600	500
T_{out} [°C]	900	1037	810	900	780/870
T_{HT-WGS} [°C]	310	310	310	310	320
T_{LT-WGS} [°C]	210	210	–	–	–
T_{PSA} [°C]	35	35	35	35	35
T_{fuel} [°C]	275	35	300	260	-
$T_{flue,in}$ [°C]	880	-	850	1100	-
$T_{flue,out}$ [°C]	130	130	180	130/180	-
$\eta_{VPSA/PSA}$ [%]	–/90	–/90	60/90	–/90	–/90
η_{CO_2} [%]	90	90	90	90	90
Excess air [%]	7	7	7	5	-
γ [%]	0.5	0.5	-	-	-

Table A.8 Summary of inlet fluxes, outlet fluxes and process metrics for LT-SMR, HT-SMR, ATR, FLOX®, PSWA and e-SMR configurations, each divided into Base and CCS cases, using Italian grid as a source of electricity.

Parameter	HT-SMR		HTLT-SMR		ATR		FLOX®		PSWA		e-SMR	
Case	Base	CCS	Base	CCS	Base	CCS	Base	CCS	Base	CCS	Base	CCS
F_{feed} $[kmol/h]$	1700	1700	1700	1700	1700	1700	0.9	0.9	22.3	22.3	11.2	11.2
α [-]	0.9	0.93	0.85	0.88	-	-	0.58	0.66	0.75	0.8	-	-
F_{O_2} $[kmol/h]$	-	-	-	-	847	847	-	-	-	-	-	-
F_{H_2} $[kmol/h]$	4569	4737	4516	4647	4591	4591	1.0	1.1	28.13	29.8	40.3	44.5
F_{CO_2} $[kmol/h]$	-	1066	-	1171	-	1561	-	0.6	-	6.6	-	11.1
$\dot{V}_{w,process}$ $[m^3/h]$	44.64	45.99	31.18	46.28	47.07	31.18	0.01	0.01	0.29	0.31	0.35	0.40
$\dot{V}_{cw}$ $[m^3/h]$	949	949	949	949	949	949	0.7	0.7	69.1	70.2	36.4	24.8
$\dot{W}_{process}$ $[MW]$	11.3	2.1	11.8	2.5	6.4	-2.3	-0.01	-0.01	-0.04	-0.2	-2.0	-1.7
η [%]	80.31	80.83	79.56	79.41	81.27	77.26	56.30	65.41	67.77	68.62	59.76	71.44
CFP $[kg_{CO_2}/kg_{H_2}]$	8.68	3.42	8.77	2.98	8.63	1.37	1.04	-9.74	0.18	-4.06	12.53	5.06

Table A.9 Summary of the parameters used for the techno-economic validation of SMR and ATR processes (η_{LHV}, CFP, Validation LCOH) against previous works

[29,36].

Parameter		η_{LHV} [%]		$CFP\,[kg_{CO_2}/kg_{H_2}]$		Validation LCOH [€/kg$_{H_2}$]	
**Work**		**This work**	**Ref.[36]**	**This work**	**Ref.[36]**	**This work**	**Ref.[29]**
HT-SMR	Base	77.5	77.1	8.7	9.0	1.57	1.68
	CCS	80.3	77.8	3.4	4.0	1.70	1.89
HT+LT-SMR	Base	76.6	76.1	8.8	9.0	-	-
	CCS	78.8	77.3	3.0	3.1	-	-
ATR	Base	77.8	76.6	8.6	9.0	-	-
	CCS	77.8	76.6	1.4	1.3	-	-

Table A.10 Summary of yearly costs, electricity costs and LCOH for HT-SMR, HTLT-SMR, ATR, FLOX®, PSWA and e-SMR configurations, each divided into Base and CCS cases.

Parameter	HT-SMR		HTLT-SMR		ATR		PSWA		e-SMR	
	**Base**	**CCS**	**Base**	**CCS**	**Base**	**CCS**	**Base**	**CCS**	**Base**	**CCS**
**C_{fuel} [M€/y]**	99.22	99.22	99.22	99.22	99.22	99.22	1.16	1.16	0.63	0.63
**OPEX [M€/y]**	12.2	12.2	13.1	13.2	12.9	12.9	0.36	0.36	0.20	0.16
**CAPEX [M€/y]**	7.80	11.92	8.20	12.71	8.39	14.28	0.24	0.26	0.37	0.32
**$CO2_{profit}$ [M€/y]**	0	29.60	0	32.50	0	42.10	0	0.20	0	0.31
**$C_{el,CH}$ [M€/y]**	-8.33	-1.55	-8.7	-1.84	-4.7	1.67	0.03	0.15	1.49	1.24
**$C_{el,DE}$ [M€/y]**	-8.10	-1.50	-8.45	-1.78	-4.57	1.62	0.03	0.15	1.45	1.20
**$C_{el,FR}$ [M€/y]**	-6.47	-1.20	-6.75	-1.42	-3.65	1.29	0.02	0.12	1.16	0.96
**$LCOH_{CH}$ [€/kg$_{H_2}$]**	1.54	1.28	1.57	1.29	1.60	1.26	4.04	3.81	4.22	3.05
**$LCOH_{DE}$ [€/kg$_{H_2}$]**	1.54	1.29	1.57	1.29	1.60	1.25	4.04	3.81	4.16	3.00
**$LCOH_{FR}$ [€/kg$_{H_2}$]**	1.54	1.29	1.60	1.30	1.61	1.25	4.02	3.75	3.70	2.65

Additional equations

Additional chemical reactions utilized in this study.

$$CH_4 + 2\,O_2 \longrightarrow CO_2 + 2\,H_2O \tag{A.55}$$

$$CO + \frac{1}{2}O_2 \longrightarrow CO_2 \tag{A.56}$$

$$H_2 + \frac{1}{2}O_2 \longrightarrow H_2O \tag{A.57}$$

How to reproduce the calculations

This section provides a concise description of the modeling and simulation procedures adopted in this work, with the aim of ensuring full reproducibility of the results. The core of the methodology relies on the correct definition and solution of material and energy balances for each plant configuration, consistently with the assumptions detailed throughout Section 2 and the parameters reported in Table 6 and Table A.7. Figures 1–6 support the visualization of the plant layouts and the associated parametric assumptions. The design and placement of heat recovery sections and heat exchangers should be independently verified by the reader through pinch analysis. Following the successful resolution of material and energy balances, plant efficiency (η) and hydrogen carbon footprint (CFP) are evaluated by substituting the resulting stream values into Equations 21–22. For reference and comparison purposes, Table A.8 reports the results obtained by the authors. The material flow rates and electrical duties derived from the mass and energy balances constitute the basis for the subsequent economic analysis. By applying the methodology and assumptions described in Section 2.6.3, together with the adopted prices summarized in Table A.13, the static levelized cost of hydrogen (LCOH) is calculated using Equation 44. Corresponding cost breakdowns and final results for each country-specific scenario are reported in Tables A.10–A.11. The dynamic LCOH assessment methodology is described in Section 2.6.3, while the associated results are presented in Table A.12. Due to the confidential

Table A.11 Comparison of Levelized Cost of Hydrogen (LCOH) and Carbon Footprint (CFP) for AWE and Methane Pyrolysis configurations across different countries (CH, DE, FR).

Technology	LCOH [€/kg$_{H_2}$]			CFP [kg$_{CO_2}$/kg$_{H_2}$]		
**Country**	**CH**	**DE**	**FR**	**CH**	**DE**	**FR**
**AWE**	6.6	6.6	5.4	18.0	2.2	1.8
**Methane Pyrolysis**	6.7	6.7	5.4	3.2	0.4	0.3

Table A.12 Summary of the dynamic and overall LCOH for each electrified technology and scenario.

Parameter	Optimal LCOH [€/kg_{H_2}]			Optimal hours [%]			Overall LCOH [€/kg_{H_2}]		
**Country**	**CH**	**DE**	**FR**	**CH**	**DE**	**FR**	**CH**	**DE**	**FR**
**e-SMR**	4.01	3.80	3.39	69.3	65.23	60.12	4.22	4.16	3.70
**e-SMR CCS**	2.94	2.79	2.50	77.5	78.14	73.3	3.05	3.0	2.65

Table A.13 Summary of the main price assumptions adopted within the work.

Parameter	Value	Reference
Natural gas cost	32 €/MWh	[78]
Biogas cost	60 €/MWh	[79]
Methane cost	32 €/MWh	[78]
Cooling water cost	0.35 €/m^3	[38]
Process water cost	2 €/m^3	[38]
HDS catalyst cost	319 €/ft^3	[38]
SMR catalyst cost	10000 €/m^3	[38]
WGS catalyst cost	2800 €/m^3	[38]
CCS membrane cost	500 €/m^2	[63]
Carbon credits	80 €/t_{CO_2}	[80]

nature of the underlying data, raw electricity price time series cannot be disclosed within the manuscript; instead, processed median monthly electricity prices for the year 2024 in the analyzed countries are reported in Table A.14. The techno-economic assessment of alkaline water electrolysis (AWE) and methane pyrolysis (MP) is presented in Subsection 2.6.4. Table 6 can be used for the verification of the carbon footprint (CFP), while Equations 52–54 are applied for the calculation of the levelized cost of hydrogen (LCOH) for AWE. The LCOH associated with methane pyrolysis cannot be directly verified, as lower and upper bounds of the LCOH were adopted from Nuran Zaini et al.[76], and no original LCOH calculations were performed within the scope of this study.

Table A.14 Median monthly day-ahead electricity prices in 2024 for French (FR), German (DE) and Swiss (CH) grid scenarios.

Parameter	Monthly median electricity price in 2024 [€/MWh]											
**Month**	**Jan**	**Feb**	**Mar**	**Apr**	**May**	**Jun**	**Jul**	**Aug**	**Sep**	**Oct**	**Nov**	**Dec**
**CH**	87.05	69.25	73.72	67.50	76.82	54.37	47.67	59.52	85.08	88.16	127.13	119.64
**DE**	78.68	61.46	66.63	64.52	80.83	83.60	78.11	91.64	82.84	86.59	111.06	102.55
**FR**	80.95	59.81	55.48	11.51	24.08	30.11	49.65	49.60	49.17	64.43	106.71	104.11
